# Rivastigmine Templates with Antioxidant Motifs—A Medicinal Chemist’s Toolbox Towards New Multipotent AD Drugs

**DOI:** 10.3390/antiox14080921

**Published:** 2025-07-28

**Authors:** Inês Dias, Marlène Emmanuel, Paul Vogt, Catarina Guerreiro-Oliveira, Inês Melo-Marques, Sandra M. Cardoso, Rita C. Guedes, Sílvia Chaves, M. Amélia Santos

**Affiliations:** 1 Centro de Química Estrutural, Institute of Molecular Sciences, Departamento de Engenharia Química, Instituto Superior Técnico, Universidade de Lisboa, Av. Rovisco Pais 1, 1049-001 Lisboa, Portugal; inesd2@edu.ulisboa.pt (I.D.); marlene-e@hotmail.fr (M.E.); paul.vogt@etu.inp-ensiacet.fr (P.V.); 2 CNC-UC, Center for Neuroscience and Cell Biology, Universidade de Coimbra, 3004-504 Coimbra, Portugal; catarinaoliveira@cnc.uc.pt (C.G.-O.); ines.marques@cnc.uc.pt (I.M.-M.); sicardoso@fmed.uc.pt (S.M.C.); 3 Centre for Innovative Biomedicine and Biotechnology, Universidade de Coimbra, 3004-504 Coimbra, Portugal; 4 FMUC, Faculdade de Medicina, Universidade de Coimbra, 3004-504 Coimbra, Portugal; 5 Research Institute for Medicines (iMed.ULisboa), Faculdade de Farmácia, Universidade de Lisboa, 1649-003 Lisboa, Portugal; rguedes@ff.ulisboa.pt

**Keywords:** Alzheimer’s disease, multi-target drugs, rivastigmine, antioxidants, cholinesterases, amyloid-β aggregation, neuroprotection

## Abstract

A series of rivastigmine hybrids, incorporating rivastigmine fragments (RIV) and a set of different antioxidant scaffolds, were designed, synthesized, and evaluated as multifunctional agents for the potential therapy of Alzheimer’s disease (AD). In vitro bioactivity assays indicated that some compounds have very good antioxidant (radical-scavenging) activity. The compounds also displayed good inhibitory activity against cholinesterases, though the bigger-sized hybrids showed higher inhibitory ability for butyrylcholinesterase (BChE) than for acetylcholinesterase (AChE), due to the larger active site cavity of BChE. All the hybrids exhibited an inhibition capacity for self-induced amyloid-β (Aβ_1–42_) aggregation. Furthermore, cell assays demonstrated that some compounds showed capacity for rescuing neuroblastoma cells from toxicity induced by reactive oxygen species (ROS). Among these RIV hybrids, the best in vitro multifunctional capacity was found for the caffeic acid derivatives enclosing catechol moieties (**4AY5**, **4AY6**), though the Trolox derivatives (**4AY2**, **4BY2**) presented the best cell neuroprotective activity against oxidative damage. Molecular-docking studies provided structural insights into the binding modes of RIV-based hybrids to the cholinesterases, revealing key interaction patterns despite some lack of correlation with inhibitory potency. Overall, the balanced multifunctional profiles of these hybrids render them potentially promising candidates for treating AD, thus deserving further investigation.

## 1. Introduction

Alzheimer’s disease (AD) is the leading cause of dementia in the elderly worldwide, according to a recent report from 2024 [1]. Since the main risk factor in AD etiopathogenesis is age, and life expectancy is progressively increasing (from 2015 to 2050, the percentage of the world’s population aged over 60 will nearly double and attain about 2.1 billion), the incidence of this disorder will increase in parallel. Currently, more than 50 million people worldwide are living with AD, and this number will increase to 152 million by 2050 [2]. Thus, as AD gradually challenges our healthcare systems and society, the urgency of more effective therapeutics becomes paramount. Although the etiology of AD remains unclear, multiple factors and pathological processes have been shown to play significant roles in the progression of this disease, such as β-amyloid (Aβ) deposits, tau protein aggregation [3], the decline of acetylcholine (ACh) [4], oxidative stress, and bio-metal dyshomeostasis [5]. The complexity and multi-factorial nature of AD, associated with difficulties in its early diagnosis (no unequivocal premortem analysis), are believed to be the main reasons for the absence of an effective cure so far. Unfortunately, the existing conventional therapies, mainly based on targeting one single causative factor, have been shown to be ineffective for AD treatment [6]. In particular, most of the current FDA-approved (about 2 decades ago) drugs are single-target small molecules that act by inhibiting cholinesterases (e.g., donepezil, rivastigmine, and galantamine) or blocking the *N*-methyl-*D*-aspartate receptor, NMDAR (memantine) [7,8], in addition to a recent manufactured combination of memantine and donepezil (not approved by European Medicines Agency, EMA) [9]. Quite recently (2021, 2023), a second generation of drugs was approved by the FDA, namely two A*β-*directed monoclonal antibodies, mAbs, (aducanumab and lecanemab), as immunotherapy based on fibril targeting and reduction of amyloid plaques [10,11]. However, the first one was later withdrawn from the market due to doubts on its actual efficacy and safety, while the efficiency of lecanemab is still modest (leading only to amelioration of some AD symptoms). But, it cannot provide AD cure, and it is exceedingly expensive (ca USD 26,500/patient/year) [12].

Given the drawbacks and limitations of the aforementioned therapies, after decades of vigorous research in drug discovery, it became widely recognized that, to combat the multifaceted AD, there was an urgent need for a paradigm shift, from traditional single-target drugs to new multi-target drugs (MTDs), which can simultaneously interfere with multiple disease-relevant proteins and provide disease-modifying effects in AD [13,14]. Therefore, this poly-pharmacological approach, relying on the development of new affordable single small molecular entities able to modulate multiple targets involved in the most relevant AD pathological processes, has been the focus of quite intensive investigation by several research groups. The design of multitarget anti-AD drugs has been mainly based on the hybridization of relevant pharmacophores that are ultimately aimed to have additive or synergistic effects as well as acceptable safety. Among the different drug development strategies, the use of pharmacophoric moieties based on the repositioning of “old” drugs has emerged to guarantee their important roles and also to avoid/reduce new drug attritions in translational processes [15,16]. In this line, our research group and others have been focused on generating novel classes of hybrids that combine the privileged scaffold of approved acetylcholinesterase (AChE) and/or butyrylcholinesterase (BChE) inhibitor drugs (tacrine, donepezil, and rivastigmine) with other pharmacophoric moieties that, in addition to contributing to guarantee the primary action of ChE inhibition, can also confer to the hybrids relevant additional therapeutic benefits, such as the inhibition of Aβ aggregation and monoaminoxidases (MAOs), antioxidant activity, and metal chelating capacity [17,18,19,20].

**Figure 1:**
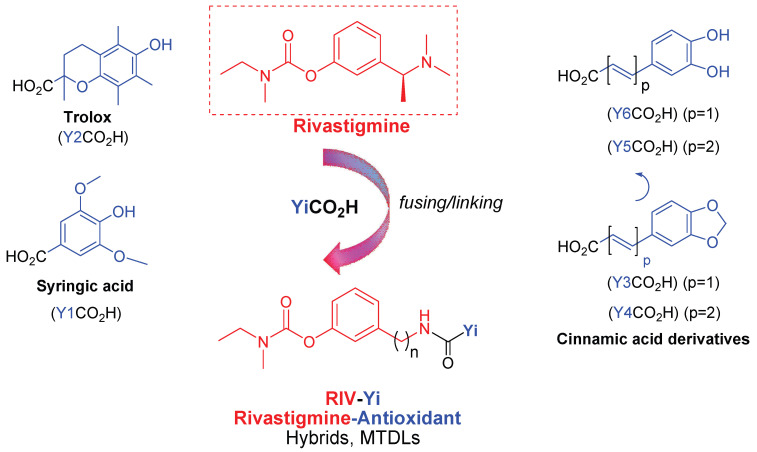
Design strategy for the rivastigmine-antioxidant hybrids.

Particularly, since the drug rivastigmine is a dual cholinesterase (AChE, BChE) inhibitor, a recognized key therapeutic target of AD (and also PD) [21], the development of rivastigmine hybrids appears to have remarkable advantages as potential anti-Alzheimer’s disease agents [22]. Therefore, pursuing with our ongoing research on rivastigmine-based hybrids [23,24] and taking into account that important recent studies revealed that oxidative stress and neuroinflammation play pivotal roles in the etiology and progression of neurodegenerative diseases [25,26,27], we developed and describe herein a series of multipotent rivastigmine hybrids (MTD) with anti-oxidant potential by fusing the active scaffold of rivastigmine (RIV) with several antioxidant (or pro-antioxidant) pharmacophoric moieties into one molecule (Figure 1), and also including some differentiation on linkers to tether these two fragments aimed at improving the binding of the hybrids with the active sites of the target proteins. For that purpose, to the RIV moiety were attached a series of carboxylic acid-enclosing moieties with recognized antioxidant (or pro-antioxidant) properties, namely Trolox, a powerful antioxidant with neuroprotective effects as an ROS scavenger and attenuator of neurotoxicity induced by Aβ and H_2_O_2_ in the brain, which has recently been fused with donepezil [28]; syringic acid, a naturally occurring polyphenol with reported neuroprotective effect, due to free radical scavenging, antioxidant capacity, anti-inflammation [29]; and dihydroxyl derivatives of cinnamic acid and cinnamylidene acetic acid, with recognized antioxidant and anti-neurodegenerative properties [30], as well as the corresponding methylenedioxy cinnamylidene acetic acid (piperic acid), a derivative of a plant metabolite (piperine) with diverse biological properties, namely as a pro-(antioxidant), which has been used in the development of diverse pharmacologically relevant compounds [31]. A set of biological activities of the compounds was evaluated, namely for their capacity to interact with some relevant targets involved in AD pathology. Therefore, all the derivatives were screened individually for their anti- AChE and BChE activities, antioxidant capacities, and inhibition of Aβ aggregation. Molecular docking and molecular dynamics simulations were carried out on the active analogs to gain insights into the binding interactions and potential structure-activity relationships. Furthermore, the neurotoxicity and neuroprotective effects of these compounds were evaluated against SH-SY5Y neuroblastoma cell lines treated with A*β*_1–42_ peptide and oxidant stressors, as in in vitro models of AD.

Therefore, the ability of these hybrids to interact with several important AD targets was analyzed, and further studies, such as inhibition of MAO activity and Cu-induced Aβ_42_ aggregation as well as biometal complexation, are on course.

## 2. Materials and Methods

### 2.1. Materials and Equipment

All reagents and solvents used were acquired with a high purity level from Sigma-Aldrich (Burlington, MA, USA), Tokyo Chemical Industry (Haven, Belgium), Honeywell Research Chemicals (Morris Plains, NJ, USA), Merck (Darmstadt, Germany), and Alfa Aesar (Lancashire, UK). The solvents, *N*,*N*-dimethylformamide (DMF) and dichoromethane (CH_2_Cl_2_), were dried according to standard methods [32]. The progress and completion of each chemical reaction were monitored by thin layer chromatography (TLC) using aluminum silica gel (60 F_254_) sheets (Macherey-Nagel Reagents, Düren, Germany) that were visualized under a UV lamp. Silica gel 60A 40–63 µm (Carlo Erba Reagents, Cornaredo (Milano), Italy) was used for flash column chromatography. Melting point (MP) measurements were made with a Leica Galen III hot-stage microscope (Leica Microsystems, Wetzlar, Germany), without temperature correction. A Bruker AVANCE III-400 NMR spectrometer (at 400 MHz and 101 MHz) and a Bruker AVANCE III-300 NMR (at 300 MHz and 75 MHz) were used to record ^1^H and ^13^C nuclear magnetic resonance (NMR) spectra (Bruker, Billerica, MA, USA). Tetramethylsilane (TMS) was used as an internal standard reference to report chemical shifts (δ) as parts per million (ppm), while the coupling constants are reported in hertz (Hz). Splitting patterns are designated by abbreviations: s (singlet), d (doublet), dd (doublet of doublets), dt (doublet of triplets), t (triplet), m (multiplet), q (quadruplet), and bs (broad singlet). Mass spectra (ESI-MS) were obtained on a 500 MS LC Ion Trap mass spectrometer (Varian Inc., Palo Alto, CA, USA) equipped with an ESI ion source, operated in the positive ion mode. High resolution mass spectra (HRMS) were obtained on a QqTOF Impact IITM mass spectrometer (Bruker Daltonics, Billerica, MA, USA) operating in the electrospray ionization (ESI) positive mode.

A Perkin Elmer Lambda 35 UV-Vis (ultraviolet–visible) spectrophotometer (PerkinElmer, Waltham, MA, USA) was used for radical scavenging and cholinesterase (ChE) inhibition studies. Fluorescence tests were conducted using a POLARstar OPTIMA microplate reader (BMG LABTECH, Ortenberg, Germany), to assess the Aβ_42_ aggregation.

### 2.2. Synthesis of the Intermediate Compounds and Final RIV Hybrids

#### 2.2.1. General Procedure for Synthesis of the Carbamates (**2A**, **2B**, and **2C**)

To a solution of triethylamine (TEA) (3 mL) were added *N*-ethyl-*N*-methyl carbamoyl chloride or dimethyl carbamoyl chloride (4.05 mmol) and the appropriate phenol derivative (3-nitrophenol or 3-cyanophenol) (3.84 mmol). The reaction mixture was left stirring at 95 °C for 22 h. Then, it was diluted with CH_2_Cl_2_ and extracted with NaOH 1M with ice and distilled water. The organic phase was dried over Na_2_SO_4_, filtrated, roto-evaporated to dryness, and then dried in vacuum to obtain the desired compound.

- 3-Nitrophenyl ethyl(methyl)carbamate (**2A**)

This product was synthesized from 3-nitrophenol and *N-*ethyl-*N-*methylcarbamoyl chloride, affording **2A** as a pale-brown oil (Yield = 92.6%). ^1^H NMR (300 MHz, MeOD-*d*_4_), **δ** (ppm): 8.09 (d, 1H, *J* = 8.0 Hz, *Ph*), 8.01 (s, 1H, *Ph*), 7.62 (t, 1H, *J* = 8.0 Hz, *Ph*), 7.54 (d, 1H, *J* = 8.0 Hz, *Ph*), 3.53 (q, 1H, *J* = 7.0 Hz, N*CH_2_*CH_3_ rotamer), 3.42 (q, 1H, *J* = 7.0 Hz, N*CH_2_*CH_3_ rotamer), 3.12 (s, 1.5H, N*CH_3_* rotamer), 3.00 (s, 1.5H, N*CH_3_* rotamer), 1.27 (t, 1.5H, *J* = 6.0 Hz, NCH_2_*CH_3_* rotamer), 1.20 (t, 1.5H, *J* = 8.0 Hz, NCH_2_*CH_3_* rotamer). MS-ESI (*m*/*z*): 225.07 (M+1)+.

- 3-Cyanophenyl ethyl(methyl)carbamate (**2B**)

This product was synthesized from 3-cyanophenol and *N*-ethyl-*N*-methylcarbamoyl chloride, affording **2B** as a pale-brown oil (Yield = 67.7%). ^1^H NMR (300 MHz, MeOD-*d*_4_), **δ** (ppm): 7.63–7.56 (m, 3H, *Ph*), 7.47 (d, 1H, *J* = 6.0 Hz, *Ph*), 3.54 (q, 1H, *J* = 7.0 Hz, N*CH_2_*CH_3_ rotamer), 3.43 (q, 1H, *J* = 7.0 Hz, N*CH_2_*CH_3_ rotamer), 3.13 (s, 1.5H, N*CH_3_* rotamer), 3.01 (s, 1.5H, N*CH_3_* rotamer), 1.29 (t, 1.5H, *J* = 7.5 Hz, NCH_2_*CH_3_* rotamer), 1.21 (t, 1.5H, *J* = 6.0 Hz, NCH_2_*CH_3_* rotamer). MS-ESI (*m*/*z*): 205.09 (M+1)+.

- 3-Nitrophenyl dimethylcarbamate (**2C**)

This product was synthesized from 3-nitrophenol and dimethyl carbamoyl chloride, affording **2C** as a white solid (Yield = 90.7%; MP = 53–55 °C). ^1^H NMR (300 MHz, MeOD-*d*_4_), **δ** (ppm): 8.13 (d, 1H, *J* = 12.0 Hz, *Ph*), 8.05 (s, 1H, *Ph*), 7.65 (t, 1H, *J* = 7.5 Hz, *Ph*), 7.57 (d, 1H, *J* = 9.0 Hz, *Ph*), 3.16 (s, 3H, N*CH_3_* rotamer), 3.03 (s, 3H, N*CH_3_* rotamer). MS-ESI (*m*/*z*): 211.06 (M+1)+.

#### 2.2.2. General Procedure for Synthesis of the Aminocarbamates (**3A**, **3B** and **3C**)

The preparation of these compounds involved a catalytic hydrogenolysis of the corresponding nitro- and cyano-carbamate derivatives (**2A**–**C**). To a solution of 3.1 mmol of each compound in 30 mL of MeOH was added 3.4 mmol of Pd-C 10% (palladium on activated carbon 10%), and this suspension was shaken in an hydrogenator flask at room temperature for 4 h under a H_2_ atmosphere (4 bar). After reaction completion (controlled by TLC), the catalyst was filtered off from the reaction mixture, and the solvent was evaporated to dryness in a vacuum. The crude product was isolated with analytical purity.

- 3-Aminophenyl ethyl(methyl)carbamate (**3A**)

This product was synthesized from 3-nitrophenyl ethyl(methyl)carbamate (**2A**), affording **3A** as a pale-brown oil (Yield = 82.4%). ^1^H NMR (300 MHz, MeOD-*d*_4_), **δ** (ppm): 7.05 (t, 1H, *J* = 7.0 Hz, *Ph*), 6.54 (d, 1H, *J* = 6.0 Hz, *Ph*), 6.42 (s, 1H, *Ph*), 6.36 (d, 1H, *J* = 6.0 Hz, *Ph*), 3.47 (q, 1H, *J* = 7.0 Hz, N*CH_2_*CH_3_ rotamer), 3.37 (q, 1H, *J* = 7.0 Hz, N*CH_2_*CH_3_ rotamer), 3.06 (s, 1.5H, N*CH_3_* rotamer), 2.95 (s, 1.5H, N*CH_3_* rotamer), 1.23 (t, 1.5H, *J* = 8.0 Hz, NCH_2_*CH_3_* rotamer), 1.16 (t, 1.5H, *J* = 8.0 Hz, NCH_2_*CH_3_* rotamer). MS-ESI (*m*/*z*): 195.06 (M+1)^+^.

- 3-(Aminomethyl)phenyl ethyl(methyl)carbamate (**3B**)

This product was synthesized from 3-cyanophenyl ethyl(methyl)carbamate (**2B**), affording **3B** as a pale-yellow oil (Yield = 75.5%). TLC was used to control the completion of the reaction (eluent: CH_2_Cl_2_/MeOH, 60/1). ^1^H NMR (400 MHz, MeOD-*d*_4_), **δ** (ppm): 7.37 (t, 1H, *J* = 8.0 Hz, *Ph*), 7.23 (d, 1H, *J* = 8.0 Hz, *Ph*), 7.13 (s, 1H, *Ph*), 7.02 (d, 1H, *J* = 8.0 Hz, *Ph*), 3.84 (s, 2H, Ph*CH_2_*NH_2_), 3.53 (q, 1H, *J* = 6.7 Hz, N*CH_2_*CH_3_ rotamer), 3.42 (q, 1H, *J* = 6.7 Hz, N*CH_2_*CH_3_ rotamer), 3.12 (s, 1.5H, N*CH_3_* rotamer), 3.00 (s, 1.5H, N*CH_3_* rotamer), 1.29 (t, 1.5H, *J* = 6.0 Hz, NCH_2_*CH_3_* rotamer), 1.20 (t, 1.5H, *J* = 6.0 Hz, NCH_2_*CH_3_* rotamer). MS-ESI (*m*/*z*): 209.04 (M+1)^+^.

- 3-Aminophenyldimethylcarbamate (**3C**)

This product was synthesized from 3-nitrophenyl dimethylcarbamate (**2C**), resulting in **3C** as a white solid (Yield = 88.5%; MP = 83–85 °C). ^1^H NMR (300 MHz, MeOD-*d*_4_), **δ** (ppm): 7.07 (t, 1H, *J* = 7.5 Hz, *Ph*), 6.57 (d, 1H, *J* = 9.0 Hz, *Ph*), 6.44 (s, 1H, *Ph*), 6.39 (d, 1H, *J* = 9.0 Hz, *Ph*), 3.10 (s, 3H, N*CH_3_* rotamer), 2.99 (s, 3H, N*CH_3_* rotamer). MS-ESI (*m*/*z*): 181.05 (M+1).

#### 2.2.3. Synthesis of (2*E*,4*E*)-5-(3,4-Dihydroxyphenyl)penta-2,4-dienoic Acid (Y5CO_2_H)

To prepare this product, 5.6 mmol of Bu_4_NI and 2.9 mmol of 5-(1,3-benzodioxol-5-yl)-2,4-pentadienoic acid (**Y4CO_2_H**) were added to 20 mL of dry CH_2_Cl_2_. The mixture was cooled to −78 °C and stirred under a nitrogen atmosphere, and then, 22 mL of BCl_3_ 1 M CH_2_Cl_2_ solution was added dropwise. After stirring for 10 min at this temperature, the solution was allowed to reach room temperature and stirred for additional 2 h. Afterwards, ice water was added to stop the reaction, and the mixture was left stirring for another 30 min. The obtained precipitate was filtered and recrystallized from CH_3_CN, providing (2*E*,4*E*)-5-(3,4-dihydroxyphenyl)penta-2,4-dienoic acid (**Y5CO_2_H**) as a pure yellow solid (Yield = 22.6%, MP = 197–199 °C). ^1^H NMR (400 MHz, MeOD-*d*_4_), **δ** (ppm): 7.42 (dd, 1H, *J* = 14.0 Hz, *J* = 10.0 Hz, COCH*CH*CHCH), 7.00 (s, 1H, *Ph*), 6.88 (t, 1H, *J* = 6.0 Hz, COCHCH*CHC*H), 6.83 (d, 1H, *J* = 4.0 Hz, COCHCHCH*CH*), 6.77 (t, 2H, *J* = 8.0 Hz, *Ph*), 5.92 (d, 1H, *J* = 16.0 Hz, CO*CH*CHCHCH). MS-ESI (*m*/*z*): 207.07 (M+1)^+^.

#### 2.2.4. Synthetic Procedures for the Target RIV Hybrids

The preparation of the final hybrid compounds involved the condensation of the amine carbamates (**3A**–**C**) with six carboxylic acids (**YiCO_2_H**, i = 1–6), using two alternative peptide coupling agents, namely *o*-benzotriazol-1-yl-*N*,*N*,*N*’,*N*’-tetramethyluronium tetrafluoroborate (TBTU) (method A) or (*N*,*N*’-dicyclohexylcarbodiimide) (DCC) (method B).

##### General Procedure (Method A) for the Synthesis of the RIV Hybrids (**4AY1**, **4AY2**, **4AY3**, **4AY4**, **4BY1**, **4BY3**, **4BY2**, and **4CY1**)

To a solution of 2.6 mmol of a carboxylic acid (**YiCO_2_H**, i =1–4) in water-ice cooled dry DMF (10 mL) under a nitrogen atmosphere, 2.99 mmol of TBTU and 5.2 mmol of NMM (*N*-methylmorpholine) were added. This reaction mixture was stirred for 50 min. Subsequently, this reaction solution (containing the activated carboxylic acid) was dropwise added to a water-ice cooled solution of 2.6 mmol of the amine carbamate (**3A**–**C**) in dry DMF (10 mL), under nitrogen atmosphere. The reaction mixture is left stirring for 20 h. TLC was used to control the completion of the reaction. At the end of the reaction, DMF was evaporated by distillation under a high vacuum. Then, the residue was diluted with EtOAc, and extracted with NaHCO_3_ (5%) solution and distilled water. The organic phase was dried over Na_2_SO_4_, filtrated off, and the solvent was evaporated to dryness. The crude products were generally purified by flash column chromatography.

- 3-(4-Hydroxy-3,5-dimethoxybenzamido)phenyl ethyl(methyl)carbamate (**4AY1**)

This product was synthesized from 3-aminophenyl ethyl(methyl)carbamate (**3A**) and 4-hydroxy-3,5-dimethoxybenzoic acid (**Y1CO_2_H**). Flash column chromatography was used to purify the obtained solid (eluent: CH_2_Cl_2_/MeOH, 4/1), providing **4AY1** as a white solid (Yield = 61.2%; MP = 169–172 °C). ^1^H NMR (400 MHz, MeOD-*d*_4_), **δ** (ppm): 7.61 (s, 1H, *Ph*), 7.55 (d, 1H, *J* = 8.0 Hz, *Ph*), 7.37 (t, 1H, *J* = 10.0 Hz, *Ph*), 7.33 (s, 2H, *Ph*), 6.90 (d, 1H, *J* = 8.0 Hz, *Ph*), 3.95 (s, 6H, PhO*CH_3_*), 3.54 (q, 1H, *J* = 5.3 Hz, N*CH_2_*CH_3_ rotamer), 3.43 (q, 1H, *J* = 8 Hz, N*CH_2_*CH_3_ rotamer), 3.13 (s, 1.5H, N*CH_3_* rotamer), 3.01 (s, 1.5H, N*CH_3_* rotamer), 1.30 (t, 1.5H, *J* = 6.0 Hz, NCH_2_C*H_3_* rotamer), 1.21 (t, 1.5H, *J* = 6.0 Hz, NCH_2_C*H_3_* rotamer). ^13^C NMR (75 MHz, MeOD-*d*_4_), **δ** (ppm): 167.07, 154.13, 151.64, 147.63, 139.78, 139.35, 128.90, 124.55, 117.53, 117.21, 114.32, 105.1, 55.51, 43.79, 33.18, 11.23. HRMS (ESI) calculated for C_19_H_23_N_2_O_6_ [M+H] 375.1556, found 375.1551.

- 3-(6-Hydroxy-2,5,7,8-tetramethylchromane-2-carboxamido)phenyl ethyl(methyl)carbamate (**4AY2**)

This product was synthesized from 3-aminophenyl ethyl(methyl)carbamate (**3A**) and 6-hydroxy-2,5,7,8-tetramethylchromane-2-carboxylic acid (**Y2CO_2_H**). Flash column chromatography was used to purify the obtained solid (eluent: CH_2_Cl_2_/MeOH, 19/1), providing **4AY2** as a pale-white solid (Yield = 37%; MP = 63–65 °C). ^1^H NMR (400 MHz, MeOD-*d*_4_), **δ** (ppm): 7.47 (s, 1H, *Ph*), 7.32–7.25 (m, 2H, *Ph*), 6.86 (d, 1H, *J* = 8.0 Hz, *Ph*), 3.50 (q, 1H, *J* = 6.7 Hz, N*CH_2_*CH_3_ rotamer), 3.39 (q, 1H, *J* = 6.7 Hz, N*CH_2_*CH_3_ rotamer), 3.09 (s, 1.5H, N*CH_3_* rotamer), 2.98 (s, 1.5H, N*CH_3_* rotamer), 2.70–2.57 (m, 2H, OCCH_2_*CH_2_*), 2.43–2.38 (m, 1H, OC*CH_2_*CH_2_), 2.27 (s, 3H, Ph*CH_3_*), 2.19 (s, 3H, Ph*CH_3_*), 2.08 (s, 3H, Ph*CH_3_*), 1.96–1.89 (m, 1H, OC*CH_2_*CH_2_), 1.58 (s, 3H, C*CH_3_*), 1.26 (t, 1.5H, *J* = 8.0 Hz, NCH_2_*CH_3_* rotamer), 1.19 (t, 1.5H, *J* = 8.0 Hz, NCH_2_*CH_3_* rotamer). ^13^C NMR (101 MHz, MeOD-*d*_4_), **δ** (ppm): 173.71, 154.77, 151.64, 146.03, 144.05, 138.4, 129.07, 121.59, 120.98, 117.56, 117.48, 117.28, 116.78, 113.53, 77.96, 43.79, 33.14, 29.42, 22.94, 20.09, 14.06, 11.44, 10.86, 10.45. HRMS (ESI) calculated for C_24_H_31_N_2_O_5_ [M+H] 427.2223, found 427.2227.

- (*E*)-3-(3-(Benzo[*d*][1,3]dioxol-5-yl)acrylamido)phenyl ethyl(methyl)carbamate (4AY3)

This product was synthesized from 3-aminophenyl ethyl(methyl)carbamate (**3A**) and 3,4-(methylenedioxy)cinnamic acid (**Y3CO_2_H**). Flash column chromatography was used to purify the obtained solid (eluent: CH_2_Cl_2_/MeOH, 32/1), providing 4AY3 as a pure pale-colored solid (Yield = 44.8%; MP = 146–149 °C). ^1^H NMR (300 MHz, MeOD-*d*4), **δ** (ppm): 7.62 (s, 1H, *Ph*), 7.60 (d, 1H, *J* = 15.0 Hz, COCH*CH*), 7.45 (d, 1H, *J* = 9.0 Hz, *Ph*), 7.35 (t, 1H, *J* = 9.0 Hz, *Ph*), 7.17 (s, 1H, *Ph*), 7.11 (d, 1H, *J* = 6.0 Hz, *Ph*), 6.89 (d, 2H, *J* = 9.0 Hz, *Ph*), 6.62 (d, 1H, *J* = 15.0 Hz, CO*CH*CH), 6.03 (s, 2H, O*CH_2_*O), 3.54 (q, 1H, *J* = 7.0 Hz, N*CH_2_*CH_3_ rotamer), 3.43 (q, 1H, *J* = 7.0 Hz, N*CH_2_*CH_3_ rotamer), 3.13 (s, 1.5H, N*CH_3_* rotamer), 3.01 (s, 1.5H, N*CH_3_* rotamer), 1.30 (t, 1.5H, *J* = 7.5 Hz, NCH_2_*CH_3_* rotamer), 1.21 (t, 1.5H, *J* = 7.5 Hz, NCH_2_*CH_3_* rotamer). ^13^C NMR (101 MHz, MeOD-*d*_4_), **δ** (ppm): 165.71, 154.85, 151.71, 148.56, 148.41, 142.29, 139.70, 130.92, 129.00, 124.42, 122.81, 122.63, 116.39, 113.28, 108.00, 105.38, 101.36, 43.78, 32.87, 11.21. HRMS (ESI) calculated for C_20_H_21_N_2_O_5_ [M+H] 369.1442, found 369.1445.

- 3-((2*E*,4*E*)-5-(Benzo[*d*][1,3]dioxol-5-yl)penta-2,4-dienamido)phenyl ethyl(methyl)carbamate (4AY4)

This product was synthesized from 3-aminophenyl ethyl(methyl)carbamate (**3A**) and 5-(1,3-benzodioxol-5-yl)-2,4-pentadienoic acid (**Y4CO_2_H**). Flash column chromatography was used to purify the obtained solid (eluent: CH_2_Cl_2_/MeOH, 32/1), providing **4AY4** as a yellow solid (Yield = 74.3%; MP = 80–82 °C). ^1^H NMR (300 MHz, MeOD-*d*_4_), **δ** (ppm): 7.60 (s, 1H, *Ph*), 7.48–7.42 (m, 2H, COCH*CH*CHCH, *Ph*), 7.34 (t, 1H, *J* = 9.0 Hz, *Ph*), 7.14 (s, 1H, *Ph*), 7.02 (d, 1H, *J* = 6.0 Hz, *Ph*), 6.92–6.82 (m, 4H, *Ph*; COCHCH*CH*CH; COCHCHCH*CH*), 6.27 (d, 1H, *J* = 15.0 Hz, CO*CH*CHCHCH), 6.00 (s, 2H, O*CH_2_*O), 3.54 (q, 1H, *J* = 7.0 Hz, N*CH_2_*CH_3_ rotamer), 3.43 (q, 1H, *J* = 7.0 Hz, N*CH_2_*CH_3_ rotamer), 3.13 (s, 1.5H, N*CH_3_* rotamer), 3.01 (s, 1.5H, N*CH_3_* rotamer), 1.29 (t, 1.5H, *J* = 6.0 Hz, NCH_2_*CH_3_* rotamer), 1.21 (t, 1.5H, *J* = 6.0 Hz, NCH_2_*CH_3_* rotamer). ^13^C NMR (75 MHz, MeOD-*d*_4_), **δ** (ppm): 165.70, 155.84, 151.68, 148.55, 148.39, 142.29, 139.71, 134.05, 130.90, 129.02, 124.40, 122.78, 122.66, 116.97, 116.37, 113.18, 108.00, 105.36, 101.36, 43.78, 32.87, 12.03. HRMS (ESI) calculated for C_22_H_23_N_2_O_5_ [M+H] 395.1608, found 395.1601.

- 3-((4-Hydroxy-3,5-dimethoxybenzamido)methyl)phenyl ethyl(methyl)carbamate (**4BY1**)

This product was synthesized from 3-(aminomethyl)phenyl ethyl(methyl)carbamate (**3B**) and 4-hydroxy-3,5-dimethoxybenzoic acid (**Y1CO_2_H**). Flash column chromatography was used to purify the obtained crude solid (eluent: AcOEt/*n*-hexane, 5/1), providing **4BY1** as a white solid (Yield = 49%; MP = 152–154 °C). ^1^H NMR (300 MHz, MeOD-*d*_4_), **δ** (ppm): 7.36 (t, 1H, *J* = 9.0 Hz, *Ph*), 7.25–7.22 (m, 3H, *Ph*), 7.12 (s, 1H, *Ph*), 7.01 (d, 1H, *J* = 6.0 Hz, *Ph*), 4.59 (s, 2H, Ph*CH_2_*), 3.90 (s, 6H, PhO*CH_3_*), 3.51 (q, 1H, *J* = 7.0 Hz, N*CH_2_*CH_3_ rotamer), 3.40 (q, 1H, *J* = 6.0 Hz, N*CH_2_*CH_3_ rotamer), 3.10 (s, 1.5H, N*CH_3_* rotamer), 2.98 (s, 1.5H, N*CH_3_* rotamer), 1.26 (t, 1.5H, *J* = 7.5 Hz, NCH_2_*CH_3_* rotamer), 1.18 (t, 1.5H, *J* = 6.0 Hz, NCH_2_*CH_3_* rotamer). ^13^C NMR (101 MHz, MeOD-*d*_4_), **δ** (ppm): 168.35, 154.96, 151.58, 147.59, 140.74, 138.96, 129.03, 124.29, 124.02, 120.67, 120.18, 104.68, 55.42, 43.75, 42.83, 33.14, 12.02. HRMS (ESI) calculated for C_20_H_25_N_2_O_6_ [M+H] 389.1711, found 389.1707.

- 3-((6-Hydroxy-2,5,7,8-tetramethylchromane-2-carboxamido)methyl)phenyl ethyl(methyl)carbamate (**4BY2**)

This product was synthesized from 3-(aminomethyl)phenyl ethyl(methyl)carbamate (**3B**) and 6-hydroxy-2,5,7,8-tetramethylchromane-2-carboxylic acid (**Y2CO_2_H**). Flash column chromatography was used to purify the obtained crude solid (eluent: CH_2_Cl_2_/MeOH, 32/1), providing **4BY2** as a white solid (Yield = 60.4%; MP = 53–55 °C). ^1^H NMR (300 MHz, MeOD-*d*_4_), **δ** (ppm): 7.17 (t, 1H, *J* = 7.5 Hz, *Ph*), 6.91 (d, 1H, *J* = 6.0 Hz, *Ph*), 6.82 (d, 1H, *J* = 9.0 Hz, *Ph*), 6.72 (d, 1H, *J* = 6.0 Hz, *Ph*), 4.48 (d, 1H, *J* = 15.0 Hz, Ph*CH_2_* rotamer), 4.24 (d, 1H, *J* = 15.0 Hz, Ph*CH_2_* rotamer), 3.49 (q, 1H, *J* = 7.0 Hz, N*CH_2_*CH_3_ rotamer), 3.39 (q, 1H, *J* = 7.0 Hz, N*CH_2_*CH_3_ rotamer), 3.09 (s, 1.5H, N*CH_3_* rotamer), 2.97 (s, 1.5H, N*CH_3_* rotamer), 2.63–2.37 (m, 4H, OC*CH_2_CH_2_*), 2.15–2.08 (m, 9H, Ph*CH_3_*), 1.54 (s, 3H, Ph*CH_3_*), 1.25 (t, 1.5H, *J* = 6.0 Hz, NCH_2_*CH_3_* rotamer), 1.18 (t, 1.5H, *J* = 6.0 Hz, NCH_2_*CH_3_* rotamer). ^13^C NMR (101 MHz, MeOD-*d*_4_), **δ** (ppm): 175.62, 155.02, 151.43, 145.84, 144.39, 140.21, 129.10, 128.97, 123.30, 121.66, 120.89, 120.07, 119.90, 117.44, 77.95, 43.80, 41.88, 29.68, 23.90, 20.33, 12.05, 11.45, 11.26, 10.75, 10.50. HRMS (ESI) calculated for C_25_H_33_N_2_O_5_ [M+H] 441.2382, found 441.2384.

- (*E*)-3-((3-(Benzo[d][1,3]dioxol-5-yl)acrylamido)methyl)phenyl ethyl(methyl)carbamate (**4BY3**)

This product was synthesized from 3-(aminomethyl)phenyl ethyl(methyl)carbamate (**3B**) and 3,4-(methylenedioxy)cinnamic acid (**Y3CO_2_H**). Flash column chromatography was used to purify the obtained crude solid (eluent: CH_2_Cl_2_/MeOH, 30/1), providing **4BY3** as a pale-colored solid (Yield = 44.1%; MP = 122–124 °C). ^1^H NMR (300 MHz, MeOD-*d*_4_), **δ** (ppm): 7.51 (d, 1H, *J* = 15.0 Hz, COCHC*H*), 7.37 (t, 1H, *J* = 7.5 Hz, *Ph*), 7.22–7.01 (m, 5H, *Ph*), 6.85 (d, 1H, *J* = 6.0 Hz, *Ph*), 6.49 (d, 1H, *J* = 15.0 Hz, CO*CH*CH), 6.01 (s, 2H, OC*H_2_*O), 4.51 (s, 2H, Ph*CH_2_*), 3.52 (q, 1H, *J* = 7.0 Hz, N*CH_2_*CH_3_ rotamer), 3.41 (q, 1H, *J* = 7.0 Hz, N*CH_2_*CH_3_ rotamer), 3.11 (s, 1.5H, N*CH_3_* rotamer), 2.99 (s, 1.5H, N*CH_3_* rotamer), 1.27 (t, 1.5H, *J* = 6.0 Hz, NCH_2_*CH_3_* rotamer), 1.19 (t, 1.5H, *J* = 6.0 Hz, NCH_2_*CH_3_* rotamer). ^13^C NMR (75 MHz, CDCl_3_), **δ** (ppm): 165.95, 154.44, 151.79, 149.12, 148.24, 141.23, 139.65, 129.57, 129.18, 124.77, 123.94, 121.30, 121.02, 118.35, 108.55, 106.40, 101.45, 44.11, 43.51, 34.27, 12.47. HRMS (ESI) calculated for C_21_H_23_N_2_O_5_ [M+H] 383.1609, found 383.1601.

- 3-(4-Hydroxy-3,5-dimethoxybenzamido)phenyl dimethylcarbamate (**4CY1**)

This product was synthesized from 3-aminophenyl dimethylcarbamate (**3C**) and 4-hydroxy-3,5-dimethoxybenzoic acid (**Y1CO_2_H**). Flash column chromatography was used to purify the obtained solid (eluent: CH_2_Cl_2_/MeOH, 12/1), providing **4CY1** as a white solid (Yield = 36.4%; MP = 215–216 °C). ^1^H NMR (300 MHz, MeOD-*d*_4_), **δ** (ppm): 7.60 (s, 1H, *Ph*), 7.55 (d, 1H, *J* = 9.0 Hz, *Ph*), 7.39 (d, 1H, *J* = 9.0 Hz, *Ph*), 7.32 (s, 2H, *Ph*), 6.91 (d, 1H, *J* = 9.0 Hz, *Ph*), 3.95 (s, 6H, PhO*CH_3_*), 3.15 (s, 3H, N*CH_3_* rotamer), 3.02 (s, 3H, N*CH_3_* rotamer). ^13^C NMR (101 MHz, MeOD-*d*_4_), **δ** (ppm): 167.06, 155.28, 151.65, 147.61, 139.78, 139.33, 128.88, 124.61, 117.52, 117.19, 114.32, 105.05, 55.47, 35.46. HRMS (ESI) calculated for C_18_H_21_N_2_O_6_ [M+H] 361.1387, found 361.1394.

##### General Procedure (Method B) for the Synthesis of the RIV Hybrids (**4AY5**, **4AY6**)

To a solution of 1.2 mmol of 3-aminophenyl ethyl(methyl)carbamate (**3A**) and 1.2 mmol of the carboxylic acid (**Y5CO_2_H** or **Y6CO_2_H**) in dry DMF (12 mL) under a nitrogen atmosphere, DCC (1.2 mmol) and *N*-hydroxysuccinimide (NHS) (1.2 mmol) were added. The reaction mixture was left stirring under a nitrogen atmosphere, at 60 °C for 6 h and then at room temperature for 15 h until completion of the reaction (monitored by TLC). The formed *N*,*N*-dicyclohexylurea (DCU) precipitate was filtered off, and DMF was evaporated under a high vacuum. The residue was taken into EtOAc and extracted with distilled water. The organic phase was dried over Na_2_SO_4_. The solid drying agent was filtered off, and the solvent was evaporated to dryness. The obtained solid compound was further purified through flash column chromatography.

- (*E*)-3-(3-(3,4-Dihydroxyphenyl)acrylamido)phenyl ethyl(methyl)carbamate (**4AY5**)

This product was synthesized from 3-aminophenyl ethyl(methyl)carbamate (**3A**) and 3,4-dihydroxycinnamic acid (**Y5CO_2_H**). Flash column chromatography was used to purify the obtained solid (eluent: CH_2_Cl_2_/MeOH, 9/1), affording **4AY5** as a pure pale-colored solid (Yield = 41.4%; MP = 88–89 °C). ^1^H NMR (400 MHz, MeOD-*d*_4_), **δ** (ppm): 7.62 (s, 1H, *Ph*), 7.55 (d, 1H, *J* = 12.0 Hz, COCH*CH*), 7.45 (d, 1H, *J* = 8.0 Hz, *Ph*), 7.34 (t, 1H, *J* = 6.0 Hz, *Ph*), 7.08 (s, 1H, *Ph*), 6.98 (d, 1H, *J* = 8.0 Hz, *Ph*), 6.87 (d, 1H, *J* = 8.0 Hz, *Ph*), 6.81 (d, 1H, *J* = 8.0 Hz, *Ph*), 6.56 (d, 1H, *J* = 16.0 Hz, CO*CH*CH), 3.53 (q, 1H, *J* = 8.0 Hz, N*CH_2_*CH_3_ rotamer), 3.43 (q, 1H, *J* = 8.0 Hz, N*CH_2_*CH_3_ rotamer), 3.13 (s, 1.5H, N*CH_3_* rotamer), 3.01 (s, 1.5H, N*CH_3_* rotamer), 1.30 (t, 1.5H, *J* = 6.0 Hz, NCH_2_*CH_3_* rotamer), 1.22 (t, 1.5H, *J* = 8.0 Hz, NCH_2_*CH_3_* rotamer). ^13^C NMR (75 MHz, MeOD-*d*_4_), **δ** (ppm): 165.97, 154.86, 151.69, 147.77, 145.42, 142.33, 139.91, 129.00, 126.75, 121.06, 117.03, 116.94, 116.34, 115.07, 113.72, 113.24, 43.78, 33.16, 12.02. HRMS (ESI) calculated for C_19_H_21_N_2_O_5_ [M+H] 357.1444, found 357.1445.

- 3-((2*E*,4*E*)-5-(3,4-Dihydroxyphenyl)penta-2,4-dienamido)phenyl ethyl(methyl)carbamate (**4AY6**)

This product was synthesized from 3-aminophenyl ethyl(methyl)carbamate (**3A**) and (2*E*,4*E*)-5-(3,4-dihydroxyphenyl)penta-2,4-dienoic acid (**Y6CO_2_H**). Flash column chromatography was used to purify the obtained solid (eluent: CH_2_Cl_2_/MeOH, 9/1), providing **4AY6** as a yellow solid (Yield = 39.3%; MP = 105–107 °C). ^1^H NMR (300 MHz, MeOD-*d*_4_), **δ** (ppm): 7.60 (s, 1H, *Ph*), 7.48–7.40 (m, 2H, *Ph*), 7.34 (t, 1H, *J* = 7.5 Hz, COCH*CH*CHCH), 7.00 (s, 1H, *Ph*), 6.92–6.76 (m, 5H, *Ph*, COCHCHCH*CH*, COCHCH*CH*CH), 6.22 (d, 1H, *J* = 15.0 Hz, CO*CH*CHCHCH), 3.54 (q, 1H, *J* = 7.0 Hz, N*CH_2_*CH_3_ rotamer), 3.42 (q, 1H, *J* = 7.0 Hz, N*CH_2_*CH_3_ rotamer), 3.13 (s, 1.5H, N*CH_3_* rotamer), 3.01 (s, 1.5H, N*CH_3_* rotamer), 1.29 (t, 1.5H, *J* = 6.0 Hz, NCH_2_*CH_3_* rotamer), 1.21 (t, 1.5H, *J* = 8.0 Hz, NCH_2_*CH_3_* rotamer). ^13^C NMR (75 MHz, MeOD-*d*_4_), **δ** (ppm): 167.76, 153.24, 151.68, 149.80, 148.33, 145.26, 140.57, 137.42, 132.57, 129.03, 128.52, 124.68, 119.99, 117.00, 116.01, 115.08, 113.19, 107.98, 43.79, 41.66. HRMS (ESI) were calculated for C_21_H_23_N_2_O_5_ [M+H] 383.1597, found 383.1602.

Figures S1–S10 in Supplementary Materials show the ^1^H NMR spectra of the final compounds (**4AY1-6**, **4BY1-3**, **4CY1**).

### 2.3. Free-Radical-Scavenging Assays

The percentage of radical-scavenging activity (%AA, antioxidant capacity) of the RIV hybrids was first determined by the 2,2-diphenyl-1-picrylhydrazyl (DPPH) method [24] using a compound concentration of 100 μM. For the compounds that showed a %AA over 50%, the value of the half maximal effective concentration (EC_50_) was calculated by preparing five methanolic solutions (5 points, 3 replicates) containing DPPH and different concentrations of the compound, up to a 3.5 mL total volume. Each calibration point was kept 30 min in darkness, and afterwards, the absorbance was measured at 517 nm. The corresponding antioxidant activity (%AA) was calculated using the equation

$$\%AA=\frac{A_{DPPH}-A_{ligand}}{A_{DPPH}}\times 100.$$

From the plot of %AA versus ligand concentration, EC_50_ was determined. Three independent experiments were performed, and a statistical analysis was conducted by a one-way analysis of variance (ANOVA) with the significance threshold set at *p* < 0.05.

### 2.4. Cholinesterase Inhibition

The enzyme inhibition of electric eel acetylcholinesterase (*ee*AChE) and equine butyrylcholinesterase (*eq*BChE) was performed according to an adaptation of Elmman’s method [30]. Stock solutions of 1 mg of each compound in 1 mL MeOH were prepared, and adequate dilutions were further made for each ligand. A control measurement (5 replicates without a ligand) and a calibration curve of 5 points with 3 replicates per point were conducted. Each solution included fixed volumes of 4-(2-hydroxyethyl)-1-piperazine-ethanesulfonic acid (HEPES) and cholinesterase (*ee*AChE or *eq*BChE), with different volumes of ligand solution. A blank solution containing HEPES and MeOH was also prepared at the same time. After 15 min of reaction, acetylcholine iodide (AChI) or butyrylcholine iodide (BChI) and 5,5’-dithiobis-(2-nitrobenzoic acid) (DTNB) were added to both solutions contained in spectrophotometer cuvettes, and the absorbance at 405 nm was recorded for 5 min (time interval = 1 s, slid width = 1 nm). The slope for each calibration point (absorbance versus reaction time) was determined, and the percent inhibition was calculated by using the equation:

$$\%Inhibition=100-(\frac{v_{i}}{v_{0}}\times 100).$$

where v_i_ is the reaction rate (slope) for each point of the calibration with the ligand, and v_0_ is the initial reaction rate (slope) for the control point (without ligand). Finally, the five-point linear regression (10–50 µL of ligand solution) corresponding to the percentage of enzyme inhibition vs. the compound concentration allowed for the determination of the half maximal inhibitory concentration (IC_50_, µM) (compound concentration for which enzyme activity is reduced to half). Experiments were performed twice for each ligand. Statistical analysis was performed by a one-way analysis of variance (ANOVA) with a significance threshold set at *p* < 0.05.

### 2.5. Inhibition of Aβ_1–42_ Self-Aggregation

Firstly, the Aβ_1–42_ peptide (Shanghai Royobiotech Co., Ltd., Shanghai, China) was treated with 1,1,1,3,3,3-hexafluoropropan-2-ol (HFIP) and then dissolved in a CH_3_CN/Na_2_CO_3_ (300 μM)/NaOH (250 μM) (48.3:48.3:4.3, *v*/*v*/*v*) mixture in order to obtain a stable stock solution (500 μM).

Solutions of 1 mg of the assayed compounds in 2 mL of 1:1 (*v*/*v*) DMSO/MeOH (0.5 mg/mL) medium were prepared and then properly diluted with phosphate buffer (0.215 M, pH 8.0) to obtain a 120 μM final concentration. The Aβ_1–42_ self-aggregation inhibition assays were made based on a reported method, involving the fluorescence emission of thioflavin T (ThT) [33,34]. So, Aβ_1–42_ (40 μM) was incubated at 37 °C for 24 h in phosphate buffer and in the presence or absence of each ligand (40 μM for all compounds but **4AY4** and **4CY1**, for which 20 μM was used). Then, 180 μM of 5 μM ThT in 50 mM glycine-NaOH (pH 8.5) buffer were added to each sample before transferring the samples to a 96-well microplate. Blank samples were also prepared without Aβ_1–42_. The ThT fluorescence was measured (446 and 485 nm excitation and emission wavelengths, respectively), and the percent of self-aggregation inhibition was determined by the equation:

I% = 100 − (IF_i_/IF_0_ × 100)

where IF_i_ and IF_0_ are the fluorescence intensities, in the presence and the absence of the assayed compound, subtracted from the fluorescence intensities due to the respective blanks. The calculated values were obtained as the mean ± SEM of four different experiments conducted in duplicate. Statistical analysis was performed by a one-way analysis of variance (ANOVA) with a significance threshold set at *p* < 0.05.

### 2.6. Molecular Modeling Studies

#### 2.6.1. Protein Structure Selection/Protein and Ligand Preparation

First, crystallographic protein structures were selected, based on the absence of mutations, co-crystallization with small molecules, and the availability of high-resolution structures [35,36]. The protein structures were retrieved from the Protein Data Bank (PDB). The selected *h*AChE crystal structures included 4EY5 [37] (in complex with (-)-huperzine A), 4EY6 [37] (in complex with (-)-galantamine), and 4EY7 [37] (in complex with donepezil). For *h*BChE, the structures chosen were 4TPK [38] (complexed with *N*-((1-(2,3-dihydro-1*H*-inden-2-yl)piperidin-3-yl)methyl)-N-(2-methoxyethyl)-2-naphthamide), 5LKR [39] (complexed with *N*-propargylpiperidines), and 7AIY [40] (complexed with 2-{1-[4-(12-amino-3-chloro-6,7,10,11-tetrahydro-7,11-methanocycloocta[*b*]quinolin-9-yl)butyl]-1*H*-1,2,3-triazol-4-yl}-*N*-[4-hydroxy-3-methoxybenzyl]acetamide).

Protein and ligand preparations were carried out using the Molecular Operating Environment (MOE) 2024.0601 software package [41]. Hydrogen atoms were added to the heavy atoms of each protein structure at pH 7.4, and the structural errors were corrected. The hydrogen bonding network was optimized to ensure that each protein structure was in its most stable and biologically relevant conformation. To reduce computational complexity, all water molecules and non-essential ligands or chains were removed [42]. Co-crystallized ligands were extracted from the respective PDB files, while the synthesized compounds were built de novo using Maestro, version 9.3 [43]. All ligands were first geometry-optimized and their ionization states adjusted to physiological pH using the Protonate-3D tool (executed in MOE 2024.0601) [44]. Multiple tautomeric forms were generated to account for potential alternative binding modes. Finally, energy minimization was performed using the Amber10:EHT force field implemented in MOE 2024.0601 to obtain a stable conformation suitable for molecular docking [42,45].

#### 2.6.2. Molecular-Docking Calculations

Self-docking and cross-docking studies were conducted using eight different scoring functions implemented across five diverse molecular-docking software packages to identify the most suitable docking strategy. The docking software and corresponding scoring functions included: Genetic Optimization for Ligand Docking (GOLD) [46] employing scoring functions such as Piecewise Linear Potential (PLP) [47], ChemScore [48], Astex Statistical Potential (ASP) [49], GoldScore [46]; MOE using London dG scoring function [41]; Generalizable Neural Network for Molecular Docking (GNINA) [50]; SMINA [51]; and GLIDE in Extra Precision (XP) mode [52,53]. To validate the reliability and predictive accuracy of each docking protocol, self-docking was performed by removing each co-crystallized ligand from its original PDB structure and re-docking it into the corresponding binding site. Additionally, cross-docking was carried out by docking each ligand into the other two selected structures of the same protein to assess the robustness across conformational variants. These docking studies allowed the selection of the most appropriate protein and scoring functions for the subsequent docking of the synthesized compounds: for *h*AChE, the optimal structure was determined to be 4EY7 in combination with the MOE London dG scoring function. For *h*BChE, the 5LKR structure paired with the GNINA scoring function was selected as the most effective. The performance of each scoring function was evaluated by calculating the root mean square deviation (RMSD) between the docked pose and the crystallographic pose. RMSD values were calculated using the fconv tool [54]. For self-docking, the RMSD values were 0.29 Å for AChE (4EY7) and 1.78 Å for BChE (5LKR), indicating excellent and acceptable pose reproduction, respectively (see Figure S11 in Supplementary Materials). As expected, the RMSD values obtained from cross-docking were higher due to structural variability among the target conformations.

The interaction profiles of the docked compounds were analyzed using the Protein–Ligand Interaction Fingerprints (ProLIF), Python library (freely installed from GitHub repository https://github.com/chemosim-lab/ProLIF, accessed on 1 June 2024) [55]. The assessed interactions included hydrogen bonds, hydrophobic interactions, and π–π stacking. As an initial validation step, the self-docked ligand poses were examined to determine whether they preserved the key interaction patterns observed in the corresponding crystallographic complexes.

### 2.7. In Vitro Cell Assays

Cell viability was assessed using the colorimetric MTT (3-(4,5-dimethylthiazol-2-yl)-2,5-diphenyltetrazolium bromide) assay, as previously described [56].

The compounds’ solutions (**4AY1-6**, **4BY1-3**, and **4CY1**) were prepared in DMSO to create stock solutions at a concentration of 25 mM, which were then stored at −20 °C. A concentration range from 0.5 µM to 20 µM was screened to identify the highest non-toxic dose. The SH-SY5Y human neuroblastoma cell line (ATCC-CRL-2266) was grown in Dulbecco’s modified Eagle’s medium (DMEM) (Thermo Fisher Scientific-Gibco, Waltham, MA, USA) supplemented with 10% heat-inactivated fetal calf serum, 50 U/mL penicillin, and 50 µg/mL streptomycin at 37 °C in a 5% CO_2_ atmosphere. The cells were seeded at a density of 0.1 × 10^6^ cells/mL (when 48-well plates were used) or 0.15 × 10^6^ cells/mL (when 24-well plates were used) one day before the experiment commenced. Following the seeding, the cells were treated with the selected concentrations of compounds for 24 h. After treatment, the cells were washed with PBS and incubated with 150 µL (in 48-well plates) or 200 µL (in 24-well plates) of MTT (0.5 mg/mL) for 2 h at 37 °C with 5% CO_2_. During this incubation, cellular dehydrogenases converted MTT into formazan crystals, which were subsequently dissolved in 150 µL (in 48-well plates) or 200 µL (in 24-well plates) of 0.04 M HCl/isopropanol, and the absorbance was measured at 570 nm.

To evaluate the compounds’ protective effects against toxicity induced by Aβ_1–42_ or iron/ascorbate, the cells were pre-treated with each compound for one hour before adding Aβ_1–42_ or iron/ascorbate, followed by an additional 24-h incubation. Aβ_1–42_ (Bachem, Torrance, CA, USA) was prepared in sterile water to create a 443 µM stock solution and was added to the cells at a final concentration of 1 µM. Ferrous sulfate and *L*-ascorbic acid (Sigma Chemical Co., St. Louis, MO, USA) were introduced into the medium at final concentrations of 5 mM and 2.5 mM, respectively. The concentrations tested to evaluate the compounds’ potential protective role were: 0.5 µM for compound **4AY5**; 1 µM for compounds **4AY2** (Aβ_1–42_), **4AY4** (Aβ_1–42_), **4AY6**, **4BY2**, and **4BY3**; 2.5 µM for compounds **4AY2** (iron/ascorbate) and **4AY4** (iron/ascorbate); 5 µM for compounds **4AY3** and **4CY1**; 10 µM for compound **4AY1**; and 15 µM for compound **4BY1**. The final concentration of DMSO did not exceed 0.05% (*v*/*v*), and no significant changes in cell morphology were observed. Each plate included control groups with untreated cells, as well as those exposed only to Aβ_1–42_ or iron/ascorbate. The ability of compounds to reduce cell viability was normalized to that of untreated controls. Data is presented as the mean ± SEM from at least three independent experiments conducted in duplicates. Statistical analysis was performed to assess normality using the Shapiro–Wilk test, followed by either a one-way analysis of variance (ANOVA) and Dunnett’s multiple comparisons post hoc test; or the Kruskal–Wallis test with Dunn’s multiple comparisons post hoc test (comparisons to tested trigger, either Aβ or Fe/Asc), with a significance threshold set at *p* < 0.05.

### 2.8. Pharmacokinetic and Physicochemical Properties

In silico prediction of the drug-like properties was performed for the newly synthesized hybrid compounds as potential therapeutic agents for AD. QikProp v.2.5 [57] was employed to estimate a range of pharmacokinetic and physicochemical parameters. These included the polar surface area (PSA), the octanol–water partition coefficient (log P), predicted binding affinity to human serum albumin (HSA), blood–brain barrier (BBB) permeability, and cellular permeability in Cancer and Madin–Darby canine kidney (MDCK) cell lines. Additionally, the potential for human oral absorption and compliance with Lipinski’s Rule of Five were assessed to evaluate drug-likeness and oral bioavailability.

## 3. Results

### 3.1. Synthetic Pathway for the RIV-IND Hybrids

**Scheme 1:**
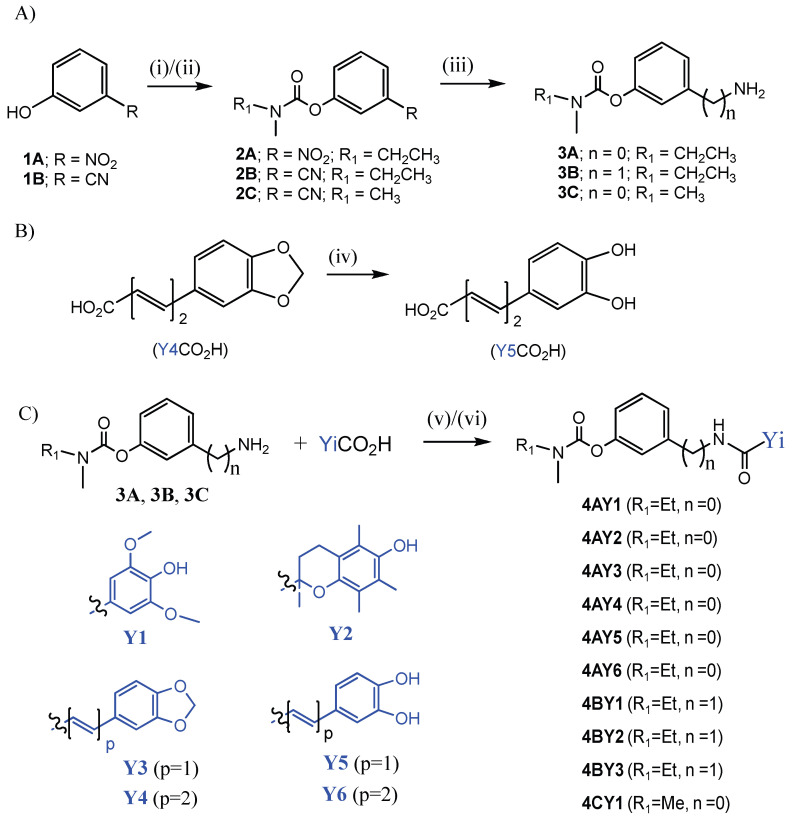
Synthesis of the target RIV hybrids (**4AY1-6**, **4BY1-3**, **4CY1**). Reaction conditions: (**A**) (i) N-ethyl-N-methylcarbamoylchloride, TEA, 95 °C, 22 h (**4AY1-6**, **4BY1-3**); (ii) Dimethylcarbamoylchloride, TEA, 95 °C, 22 h (**4CY1**); (iii) MeOH, 10% Pd-C, 4 h, H_2_, 4 bar; (**B**) (iv) BCl_3_, *n*-Bu_4_NI, dry CH_2_Cl_2_, −78 °C, N_2_; (**C**) (v) TBTU, NMM, dry DMF, 0° to rt, N_2_, 22 h (for **4AY1-4**, **4BY1-3**, **4CY1**); (vi) DCC, NHS, dry DMF, 60 °C to rt, 21h (**4AY5-6**).

The synthetic protocol used to obtain this series of ten rivastigmine hybrids (**4AY1-6**, **4BY1-3**, and **4CY1**) is summarized in Scheme 1A–C.

The synthesis of the compounds started with the preliminary preparation of the four key intermediates, namely three amino–rivastigmine (RIV) derivatives (**3A**, **3B**, **3C**, Scheme 1A) and one cinnamic acid derivative (**Y5CO_2_H**, Scheme 1B).

The synthesis of the amino–RIV intermediates involved two reaction steps, which were carried out as previously described [23,24]. Firstly, the carbamylation of the phenolic derivatives (3-nitrophenol or 3-cyanophenol) was carried out by reaction with appropriate carbamoyl chlorides *(N*-ethyl-*N*-methylcarbamoyl chloride or dimethylcarbamoyl chloride). The nitro- and nitrilo-carbamoyl derivatives (**2A**–**C**) were obtained with yields of 67–93%. These carbamate derivatives were subsequently subjected to reductive hydrogenation in MeOH with the catalyst Pd-C 10%, under an H_2_ atmosphere (4 bar) at room temperature for 4 h, affording the corresponding amino– and aminomethylene–carbamate derivatives (**3A**–**C**), with yields of 56–89%.

For the preparation of the final RIV hybrids, the primary amine-containing carbamates (amino–RIV derivatives) were condensed with several functionalized aromatic carboxylic acids. However, since one of the target carboxylic acid reagents is not commercially available, an extra preliminary reaction was performed (Scheme 1B). It involved the acetal cleavage of a methylenedioxy cinnamic moiety of the commercially available 5-(1,3-benzodioxol-5-yl)-2,4-pentadienoic acid (piperic acid, **Y4CO_2_H**) to obtain the corresponding derivative with two free phenolic hydroxyl (catechol) groups (**Y5CO_2_H**). This alkyl-aromatic ether cleavage requires very mild conditions, as previously reported [30], namely, using a mixture of the Lewis acid boron trichloride (BCl_3_) with anhydrous tetra-*n*-butylammonium iodide (*n*-Bu_4_NI) in dry CH_2_Cl_2_ under a nitrogen atmosphere and at low temperature (−78 °C), affording the final product with 23% yield.

With the key intermediates in hand, the series of ten final RIV hybrids, (**4AY1-6**, **4BY1-3**, **4CY1**) were obtained from the condensation of amino–RIV derivatives (**3A**–**C**) with six aromatic carboxylic acids (**YiCO_2_H**, i = 1–6) using two alternative standard coupling activating reagents (TBTU or DCC) under nitrogen atmosphere and dry DMF as a solvent, as depicted in reaction Scheme 1C. The final RIV hybrids (**4AY1-4**, **4BY1-3**, and **4CY1**) were obtained using TBTU, with yields of 45–86%, while for **4AY5** and **4AY6**, the milder coupling agent DCC was used, with 56–74% yields.

All the intermediates and final products were characterized by ^1^HNMR spectroscopy and ESI mass spectrometry, while the target final RIV hybrids were further characterized by ^13^CNMR spectroscopy and HRMS (ESI) mass spectrometry.

### 3.2. Free-Radical-Scavenging Evaluation

The RIV hybrids were screened for their radical-scavenging activity by using the DPPH method [24]. These assays were conducted in two stages: first, preliminary analyses were performed for all the compounds to evaluate the percentage of radical-scavenging activity by using a compound concentration of 100 μM. Afterwards, the compounds showing more than 50% antioxidant activity (AA), under those conditions, were further assessed to evaluate the respective EC_50_ values.

The data contained in Table 1 shows that compounds **4AY2**, **4AY5**, **4AY6**, and **4BY2** present a high percentage (93.1–96%) of radical-scavenging activity at 100 μM, and so, their EC_50_ (15.7–28 μM) values were subsequently determined. Meanwhile, the hybrids **4AY3**, **4AY4**, and **4BY3** have low radical-scavenging activity (<50% at 100 μM, see Table 1).

### 3.3. Inhibition of Cholinesterases

Both AChE and BChE are found ubiquitously throughout the body and possess a variety of functions dependent upon their location. Particularly in the healthy brain, AChE and BChE play, respectively, a major and a minor role in regulating brain acetyl choline (ACh). However, in patients with AD, BChE activity progressively increases, while AChE activity remains or even declines. Notwithstanding some differences in their structural features and concomitant substrate specificity, both ChE enzymes have been considered legitimate therapeutic targets for ameliorating the cholinergic deficit, and it has gained major interest regarding the development of ChE dual inhibitors as anti-AD drugs.

An analysis of the results of ChE inhibition, presented in Table 1, shows that the best inhibitory potential for AChE was found for **4AY6** (IC_50_ = 5 ± 1 µM), while the best BChE inhibition was found for **4BY3** (IC_50_ = 0.9 ± 0.2 µM). Furthermore, in the case of AChE, two compounds (**4AY4** and **4AY6**) are able to improve the enzymatic inhibition relative to the parent compound (Rivastigmine), while the racemic mixture of **4BY2** showed similar inhibitory activity (see Table S1). In the case of BChE, four compounds (**4AY3**, **4AY5**, **4AY6**, and the racemic mixture of **4BY2**) present good inhibitory potential (IC_50_ = 3.1–7.2 µM), while the inhibitory activity of **4BY3** is analogous to that of Rivastigmine (see Table S1). All the compounds, except for the hybrids **4AY4** and **4AY6**, showed moderate selectivity towards BChE, as compared with that reported for Rivastigmine, though **4BY3** presented an even higher selectivity index than the parent compound.

### 3.4. Inhibition of Self Aβ_1–42_ Aggregation

The inhibitory assays were generally performed in the presence of 40 µM of inhibitor, though half of this concentration was used for **4AY4** and **4CY1** due to solubility limitations. The obtained results, as a percentage of inhibition of Aβ_42_ aggregation, are depicted in Table 1. An analysis of these results shows that all the compounds have anti-amyloidogenic capacity, generally with very good values (>45%), though the best Aβ_1–42_ aggregation inhibition was found for two catecholate derivatives, **4AY5** and **4AY6**, analogous to curcumin (a selected reference compound with high anti-amyloidogenic activity), while the lowest value was found for **4AY1**.

### 3.5. Molecular-Docking Studies

Molecular-docking studies were performed to try to anticipate and rationalize the experimental results obtained for inhibiting both cholinesterases by the RIV-based hybrids (Section 3.3) and to identify binding interactions within the human enzyme active sites. The in vitro enzyme inhibitory assays were performed with the commercially available soluble isoforms *ee*AChE and *eq*BChE, and not with *h*AChE and *h*BChE, respectively, mainly due to financial reasons. Although eeAChE and *h*AChE share a great level of similarity (higher than 90%) and *eq*BChE differs from *h*BChE in ca 15 amino acids, there is literature evidence that, within each pair of enzymes (*ee*AChE/*h*AChE, *eq*BChE/*h*BChE), the inhibition efficiency becomes different or similar depending on the inhibitor [58,59]. Therefore, comparisons between the in vitro and the in silico enzyme inhibitory studies herein performed have to be analyzed with prudence.

**Figure 2:**
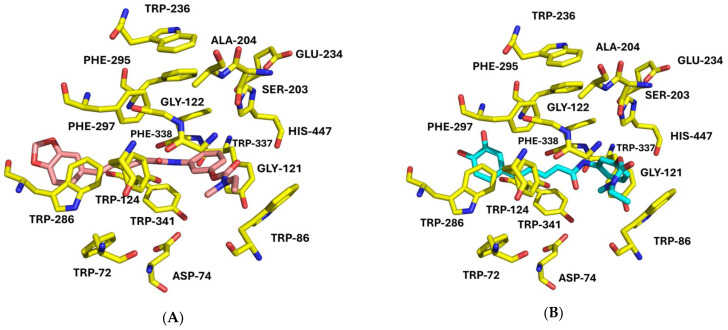
Predicted binding poses for the RIV hybrids within the active site of *h*AChE, based on the crystal structure with PDB ID: 4EY7 [37]: (**A**) compound **4AY4** (shown in salmon); (**B**) compound **4AY6** (shown in cyan).

Figure 2 illustrates the predicted binding poses of the most potent *ee*AChE inhibitors, compounds **4AY4** and **4AY6**, within the active site of hAChE. Both compounds position their RIV moiety deeply within the CAS, exhibiting similar orientations and binding modes, as well as most synthesized hybrids adopting a binding orientation comparable to **4AY4** and **4AY6**. Notably, exceptions to this trend include compound **4AY3** and both isomers of the Trolox derivatives (**4AY2** and **4BY2**), which displayed an alternative binding mode with the RIV moiety preferentially located in the PAS, as shown in Figure S12.

Although racemic mixtures consist of enantiomers with identical chemical structures, their biological activities often differ significantly. For the Trolox-based derivatives **4AY2** and **4BY2**, both enantiomeric pairs exhibited some degree of spatial overlap in their docking poses. Notably, the enantiomers of **4BY2** appear to have a higher degree of superimposition compared to those of **4AY2**, and in both cases, the RIV moiety is oriented toward the PAS.

Docking score values for the top-ranked binding poses of all compounds, along with the crystallographic ligand donepezil, were obtained using the MOE London dG scoring function and are presented in Table S2.

A further analysis of the interaction profiles of the RIV hybrids within the *h*AChE active site, generated by ProLIF [55], revealed consistent interactions with several key residues. Notably, TRP86 (hydrophobic, vdW contact), TYR337 (hydrophobic, π–π stacking), TYR341 (vdW contact), TYR124 (vdW contact), HIS447 (hydrophobic), and PHE338 (hydrophobic, vdW contact) were frequently involved in ligand binding and are also characteristic of the known inhibitor rivastigmine. In addition, most RIV hybrids (except **4AY4**, **4AY5**, and **4AY6**) shared interaction patterns with their respective parent acids. For example, the syringic acid derivatives (**4AY1**, **4BY1**, and **4CY1**) interacted with TRP286 (hydrophobic) and TYR341 (hydrophobic, vdW contact). The Trolox derivatives (**R4AY2**, **S4AY2**, **R4BY2**, and **S4BY2**) engaged TRP86 (hydrophobic, π–π stacking, and vdW contact), TYR337 (hydrophobic), and HIS447 (hydrophobic), while the piperic (**4AY3**) and cinnamic acid (**4BY3**) derivatives primarily interact with TYR337 (hydrophobic) and TYR341 (hydrophobic).

**Figure 3:**
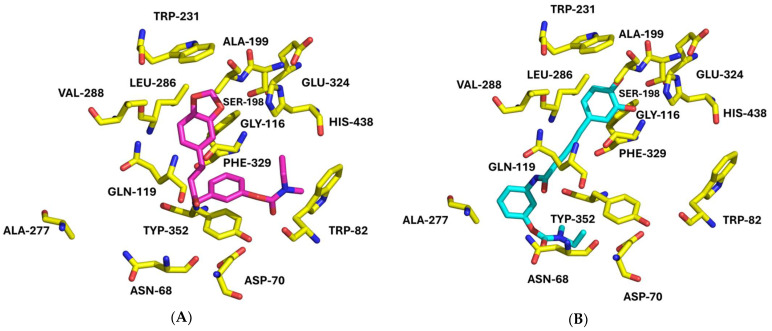
Predicted docking poses for the RIV hybrids within the active site of *h*BChE, based on the crystal structure with PDB ID: 5LKR [39]: (**A**) compound **4BY3** (shown in dark pink); (**B**) compound **4AY6** (shown in cyan).

Concerning *h*BChE inhibition, the optimal docking poses for selected inhibitors (**4BY3**, **4AY6**) are illustrated in Figure 3. Due to the larger and more flexible binding gorge of *h*BChE, the docked compounds tend to adopt a broader range of conformations, including U-shaped geometries in some cases, making the definition of a consistent binding orientation more challenging. For example, the most potent *h*BChE inhibitor identified in this study, **4BY3**, positions both its acid-derived moiety and RIV fragment close to the CAS, suggesting dual anchoring interactions that may enhance binding affinity (see Figure 3). In contrast, compound **4AY6** adopts a more extended, linear conformation, with its RIV moiety oriented to the PAS, indicating a distinct binding mode within the broader active-site gorge of *h*BChE.

For the chiral compounds **4AY2** and **4BY2**, considerable differences in binding orientation were observed between the R and S isomers. Specifically, the R enantiomer of **4AY2** adopts a binding mode more closely aligned with that of potent inhibitors. In contrast, the S enantiomer of **4BY2** occupies a spatial orientation like that of **4BY3**, the most active compound.

Docking score values obtained using the GNINA scoring function are summarized in Table S2. While the scores provide a useful, albeit imperfect, estimation of ligand–receptor binding affinity, no consistent relationship could be established between these scores and the experimental inhibitory activity of the compounds against BChE.

An interaction fingerprint analysis revealed that only three residues, SER198 (vdW contact), PHE329 (hydrophobic, vdW contact), and VAL288 (hydrophobic), were commonly engaged by the RIV hybrids and the parent drug rivastigmine. These interactions were present in all compounds except both enantiomers of **4AY2** and **4BY2**. A similar interaction pattern was observed with the original acid-derived moieties, which also interacted with SER198 and PHE329, except for **4AY2** and **4BY2**.

### 3.6. Assessment of Cell Viability and Protective Effects

**Figure 4:**
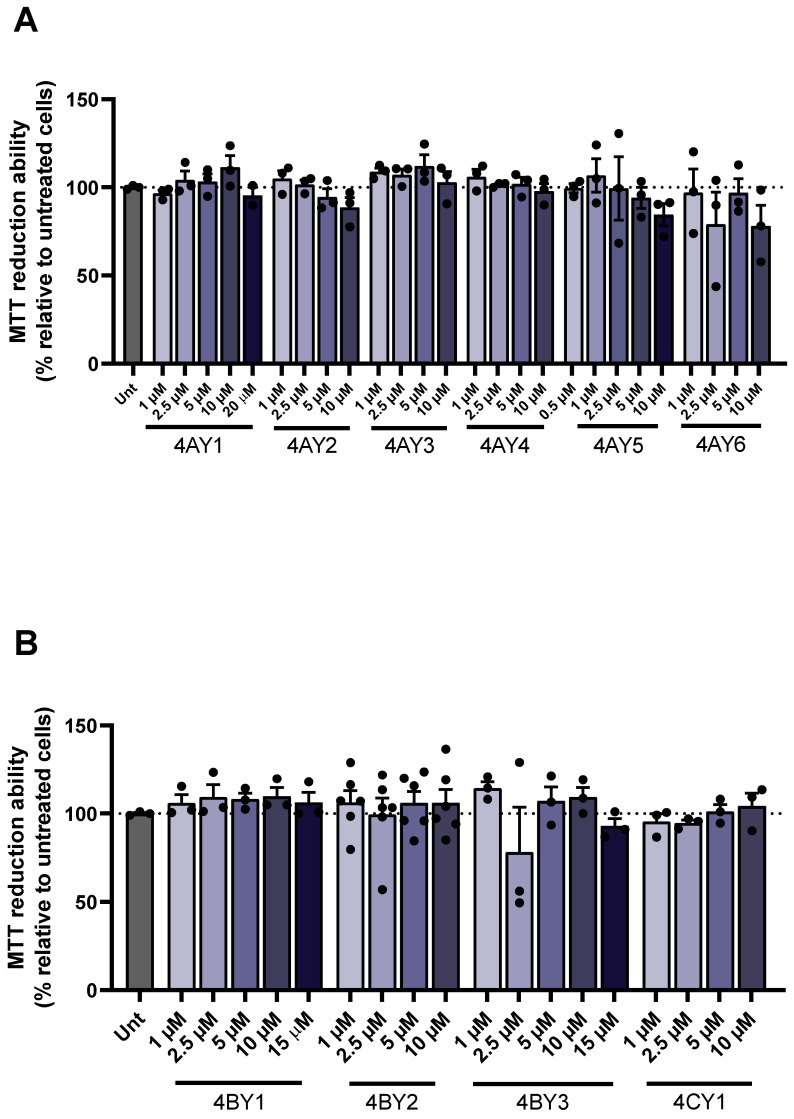
Independent single-compound dose–response screening of RIV compounds. SH-SY5Y cells were treated with compounds (**A**) **4AY1-6**, (**B**) **4BY1-3**, and **4CY1** at varying concentrations for 24 h to assess their effects on cell viability measured using the MTT reduction assay. Results are expressed relative to untreated cells, with the mean ± standard error of the mean (SEM) derived from at least three independent experiments. Compounds **4AY1**, **4AY2**, **4AY4**, **4AY5**; **4AY6**, **4BY1**, **4BY2**, **4BY3**, and **4CY1** were evaluated with ANOVA, with F-statistics parametric analysis of F(5,11) = 1.661, *p* = 0.2242; F(4,10) = 0.8752, *p* = 0.5120; F(4,10) = 2.479, *p* = 0.1114; F(4,10) = 0.8007, *p* = 0.5516; F(5,12) = 0.6780, *p* = 0.6485; F(5,12) = 0.5487, *p* = 0.7368; F(5,12) = 1.352, *p* = 0.3084; F(4,22) = 0.1960, *p* = 0.9379; F(4,10) = 0.9102, *p* = 0.4943, respectively. Compound **4AY3** was evaluated with Kruskal–Wallis non-parametric test with Dunn’s post hoc, H(4) = 4.900, *p* = 0.3262.

The RIV hybrids were tested to determine their protective role in the neuroblastoma cell toxicity induced by either Aβ_1–42_ peptide or iron/ascorbate. SH-SY5Y cells were incubated with a set of concentrations of each of the compounds, and a dose–response curve was performed to select the highest nontoxic concentration. This dose was found to be 0.5 µM for compound **4AY5**; 1 µM for compounds **4AY2** (Aβ_1–42_), **4AY4** (Aβ_1–42_), **4AY6**, **4BY2**, and **4BY3**; 2.5 µM for compounds **4AY2** (iron/ascorbate) and **4AY4** (iron/ascorbate); 5 µM for compounds **4AY3** and **4CY1**; 10 µM for compound **4AY1**; and 15 µM for compound **4BY1** (Figure 4).

**Figure 5:**
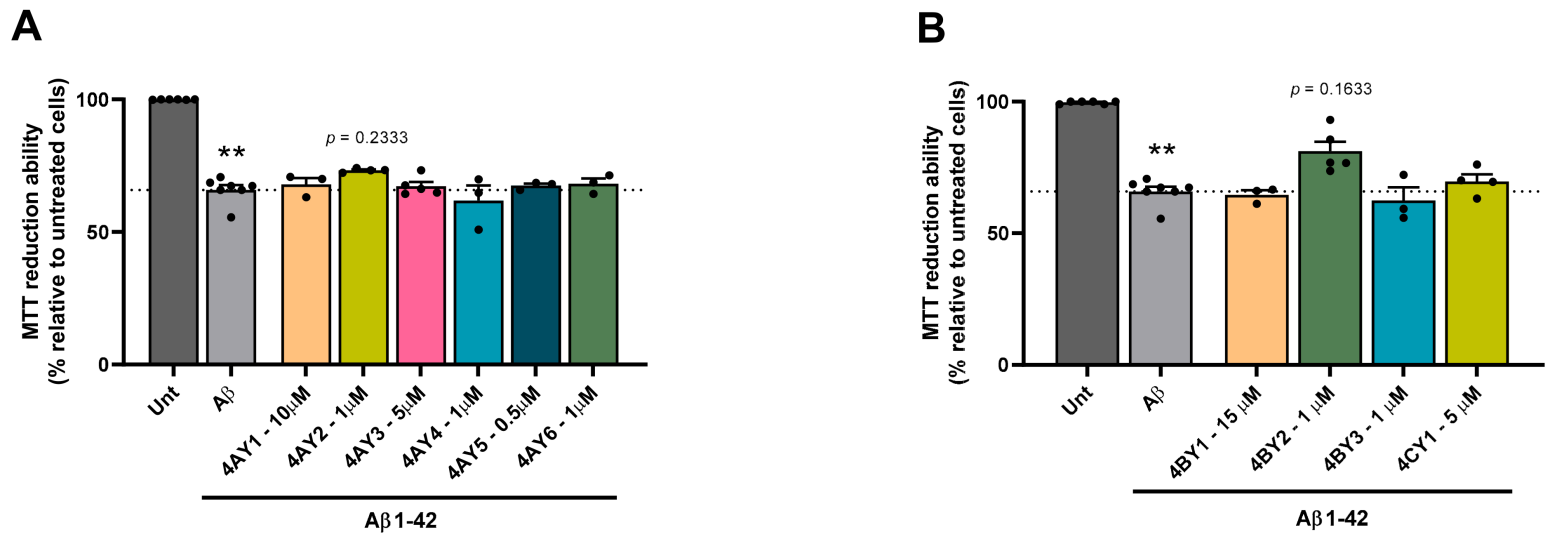
Neuroprotective effect of RIV compounds against cellular toxicity induced by Aβ_1–42_. The protective effect of the compounds (**A**) **4AY1-6** and (**B**) **4BY1-3** and **4CY1** was evaluated at a single concentration for one hour, followed by a 24 h co-incubation with Aβ_1–42_. Cell viability was assessed using the MTT reduction assay. Results are expressed relative to untreated cells, with the mean ± standard error of the mean (SEM) derived from at least three independent experiments. Statistical analysis was performed individually for each compound by comparing untreated, Aβ_1_–_42_-treated, and compound + Aβ_1_–_42_ co-treated groups. If the data were non-parametric, the Kruskal–Wallis test followed by Dunn’s post hoc test was used. If the data were parametric, one-way ANOVA followed by Dunnett’s post hoc test was applied. Test results were as follows. For 4AY1: H(2) = 13.02; *p* < 0.0001; Aβ vs. Unt: *p* = 0.0011 **; Aβ vs. 4AY1—10 μM: *p* > 0.9999. For 4AY2: H(2) = 15.83; *p* < 0.0001; Aβ vs. Unt: *p* = 0.0001 ***; Aβ vs. 4AY2—1 μM: *p* = 0.2333. For 4AY3: H(2) = 13.86; *p* < 0.0001; Aβ vs. Unt: *p* = 0.0022 **; Aβ vs. 4AY3—5 μM: *p* = >0.9999. For 4AY4: H(2) = 13.02; *p* < 0.0001; Aβ vs. Unt: *p* = 0.0039 **; Aβ vs. 4AY4—1 μM: *p* > 0.9999. For 4AY5: H(2) = 12.90; *p* < 0.0001; Aβ vs. Unt: *p* = 0.0015 **; Aβ vs. 4AY5—0.5 μM: *p* > 0.9999. For 4AY6: H(2) = 13.02; *p* < 0.0001; Aβ vs. Unt: *p* = 0.0011 **; Aβ vs. 4AY6—1 μM: >0.9999. For 4BY1: H(2) = 13.02; *p* < 0.0001; Aβ vs. Unt: *p* = 0.0039 **; Aβ vs. 4BY1—15 μM: *p* > 0.9999. For 4BY2: H(2) = 16.89; *p* < 0.0001; Aβ vs. Unt: *p* < 0.0001 ****; Aβ vs. 4BY2—1 μM: *p* = 0.1633. For 4BY3: H(2) = 12.85; *p* < 0.0001; Aβ vs. Unt: *p* = 0.0027 **; Aβ vs. 4BY3—1 μM: >0.9999. For 4CY1: H(2) = 13.82; *p* < 0.0001; Aβ vs. Unt: *p* = 0.0007 ***; Aβ vs. 4CY1—5 μM: *p* > 0.9999. ** *p* < 0.01 compared to untreated cells.

**Figure 6:**
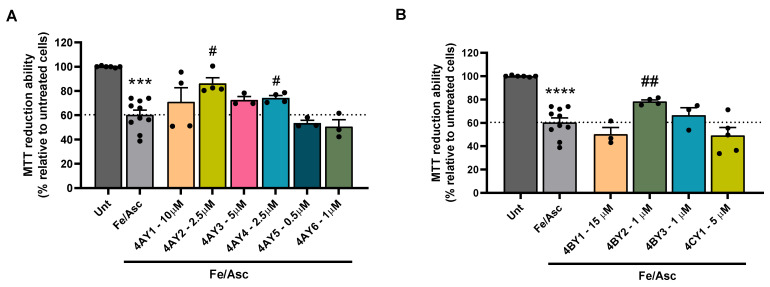
Neuroprotective effect of RIV compounds against cellular toxicity induced by iron/ascorbate (Fe/Asc). The protective effect of the compounds (**A**) **4AY1-6** and (**B**) **4BY1-3** and **4CY1** was evaluated at a single concentration for one hour, followed by a 24 h co-incubation with Fe/Asc. Cell viability was assessed using the MTT reduction assay. Results are expressed relative to untreated cells, with the mean ± standard error of the mean (SEM) derived from at least three independent experiments. Statistical analysis was performed individually for each compound by comparing untreated, Fe/Asc-treated, and compound + Fe/Asc co-treated groups. If the data were non-parametric, the Kruskal–Wallis test followed by Dunn’s post hoc test was used. If the data were parametric, one-way ANOVA followed by Dunnett’s post hoc test was applied. All comparisons were made among the three groups for each compound tested. Test results were as follows. For 4AY1: F(2,17) = 16.68; *p* < 0.0001; Fe/Asc vs. Unt: *p* <0.0001 ****; Fe/Asc vs. 4AY1—10 μM: *p* = 0.3493. For 4AY2: H(2) = 14.99; *p* < 0.0001; Fe/Asc vs. Unt: *p* = 0.0005 ***; Fe/Asc vs. 4AY2—2.5 μM: *p* = 0.0359 *. For 4AY3: H(2) = 12.93; *p* < 0.0001; Fe/Asc vs. Unt: *p* = 0.0006 ***; Fe/Asc vs. 4AY3—5 μM: *p* = 0.5802. For 4AY4: F(2,17) = 34.61; *p* < 0.0001; Fe/Asc vs. Unt: *p* < 0.0001 ****; Fe/Asc vs. 4AY4—2.5 μM: *p* = 0.0390 *. For 4AY5: F(2,16) = 39.60; *p* < 0.0001; Fe/Asc vs. Unt: *p* < 0.0001 ****; Fe/Asc vs. 4AY5—0.5 μM: *p* = 0.4822. For 4AY6: F(2,16) = 37.27; *p* < 0.0001; Fe/Asc vs. Unt: *p* < 0.0001 ****; Fe/Asc vs. 4AY6—1 μM: *p* = 0.2798. For 4BY1: F(2,16) = 37.47; *p* < 0.0001; Fe/Asc vs. Unt: *p* < 0.0001 ****; Fe/Asc vs. 4BY1—15 μM: *p* = 0.2645. For 4BY2: F(2,17) = 35.35; *p* < 0.0001; Fe/Asc vs. Unt: *p* < 0.0001 ****; Fe/Asc vs. 4BY2—1 μM: *p* = 0.0079 **. For 4BY3: F(2,16) = 29.47; *p* < 0.0001; Fe/Asc vs. Unt: *p* < 0.0001 ****; Fe/Asc vs. 4BY3—1 μM: *p* = 0.5846. For 4CY1: F(2,18) = 32.64; *p* < 0.0001; Fe/Asc vs. Unt: *p* < 0.0001 ****; Fe/Asc vs. 4CY1—5 μM: *p* = 0.1669. *** *p* < 0.001, **** *p* < 0.0001 compared to untreated cells; # *p* < 0.05 and ## *p* < 0.01 compared to Fe/Asc-treated cells.

A significant decrease in cell viability was observed following incubation with Aβ_1–42_ (Figure 5) and iron/ascorbate (Figure 6) of an average of 66% and 60%, respectively. Interestingly, none of the tested compounds were able to rescue the cell toxicity induced by Aβ_1–42_ (Figure 5). Compounds **4AY2**, **4AY4**, and **4BY2** were capable of rescuing the cell viability induced by ROS after treatment with iron/ascorbate (Figure 6).

### 3.7. Evaluation of Pharmacokinetic and Physicochemical Properties

The set of predicted properties include molecular weight (MW), van der Waals surface area of polar nitrogen and oxygen atoms (PSA), octanol/water partition coefficient (*c*log P_o/w_), binding interaction with human serum albumin (log *K*(HSA)), brain–blood partition coefficient (log BB), apparent Caco-2 cell permeability (Caco-2 permeab.), apparent MDCK (Madin–Darby canine kidney) cell permeability (MDCK permeab.), and percentage of human oral absorption.

Table 2 reveals that, generally, the compounds fall within the optimal range of values defined for each parameter. However, **4AY5** and **4AY6** exhibit suboptimal predicted Caco-2 and MDCK cell permeabilities and moderate/high percentages of human oral absorption, and **4BY1** also demonstrates an MDCK permeability value that is acceptable but not optimal.

Since it is known that enantiomers can exhibit different metabolic, toxicological, and pharmacological characteristics, the pharmacokinetic properties of the chiral compounds (**4AY2**, **4BY2**) were predicted separately for each enantiomer. While some pharmacokinetic parameters for **4AY2**’s ***R*** and ***S*** isomers are similar, the most significant discrepancy is observed in log BB, with **S4AY2** showing a 6% higher value than **R4AY2**. For **4BY2**, significant differences were noted between the enantiomers, particularly in the MDCK permeability values: **R4AY2** has excellent permeability for MDCK cells, while **S4BY2** is just within the acceptable range.

## 4. Discussion

### 4.1. Synthesis of the RIV-IND Hybrids

The synthesis pathway to obtain the ten final RIV hybrids (**4AY1-6**, **4BY1-3** and **4CY1**) was already shown in Section 3.1, Scheme 1A–C. In this procedure, the solvent used for carbamylating 3-nitrophenol or 3-cyanophenol was TEA, which also acts as a base to neutralize the HCl produced during the reaction. For most of the condensation reactions of the amino-RIV derivatives (**3A**–**C**) with the six aromatic carboxylic acids (**YiCO_2_H**, i = 1–6), TBTU was used because, based on similar reported reactions [23,24], it generally leads to fewer side reactions and better yields than DCC. However, the same procedure was not successful for the preparation of the hybrids **4AY5** and **4AY6** because a lot of side products were formed, thus making the purification of the target compounds difficult. This abnormal behavior may be attributed to the fact that the corresponding carboxylic reagents (**Y5CO_2_H** and **Y6CO_2_H**) have a catechol group, which could also react with TBTU. Therefore, in these cases, the acid–base condensation was carried out using DCC as a milder coupling activating agent, therefore obtaining better yields.

### 4.2. Free-Radical-Scavenging Activity

When assaying the radical-scavenging capacity of the RIV hybrids, it was verified that the Trolox-based hybrids (**4AY2** and **4BY2**) have EC_50_ values similar to the parent acid compound (Trolox), as already reported in the literature also for Trolox amide derivatives [60]. On the other hand, since the antioxidant capacity of phenolic acids is primarily related to the number and position of phenolic OH groups [61], compounds **4AY5** and **4AY6** showed the expected high radical-scavenging ability, like their parent compounds, caffeic acid (EC_50_ = 21.7 ± 0.2 μM [62]) and (2*E*,4*E*)-5-(3,4-dihydroxyphenyl)penta-2,4-dienoic acid (EC_50_ = 18.2 ± 0.2 μM [63]), respectively.

Regarding compounds **4AY3**, **4AY4**, and **4BY3**, their low radical-scavenging activities (<50% at 100 μM) were somehow expected due to the absence of free phenol groups, and neither piperic acid nor 3,4-(methylenedioxy)cinnamic acid are strong antioxidants [63,64].

Although syringic acid is recognized as a good antioxidant, the loss of activity of the herein developed syringic acid derivatives (**4AY1**, **4BY1**, and **4CY1**) may be attributed to the absence of carboxylic acid group in these RIV hybrids. In fact, a study on syringic acid derivatives, in which hybridization occurred at the phenolic hydroxyl group but keeping the carboxylic acid group in the final hybrids, showed that these compounds still retained some antioxidant capacity [65], thus suggesting the importance of the carboxylic groups in that activity.

### 4.3. Inhibition of Cholinesterase Activity

The evaluation of potential effects of the linker size on the ChE inhibitory activity can be made on the basis of comparison of the results obtained for **4AY1**, **4AY2**, and **4AY3**, in contrast with those for the corresponding analogues **4BY1**, **4BY2**, and **4BY3**, containing one extra methylene group between the RIV and the aromatic acid moieties. Concerning the syringic acid derivatives (**4AY1** and **4BY1**), although those effects on AChE inhibition (AChEi) could not be accessed, the shorter linker size hybrid (**4AY1**) shows higher BChE inhibitory activity (BChEi) than the corresponding analogue with longer size (**4BY1**). On the other hand, **4AY3** shows higher AChEi but lower BChEi than **4BY3**. Notably, the racemic mixture of the Trolox derivative with shorter linker size (**4AY2**) presents lower inhibitory capacity for both enzymes than that with longer size (**4BY2**). Furthermore, the slight modification in the *N*-substituent groups of the RIV moiety, namely changing a *N*-ethyl (**4AY1**) with a *N*-methyl group (**4CY1**), resulted in a lower BChEi, though both hybrids present relatively low AChEi activity.

On the other hand, the effect of adding an extra alkene group in the aromatic acid moiety in the two pairs of hybrids (**4AY4** and **4AY6**, compared to **4AY3** and **4AY5**, respectively) was analyzed. The results evidenced that this additional group consistently increased the AChEi, though with a more pronounced effect in the pair of the catechol-containing hybrids (**4AY5**, **4AY6**). Concerning the BChEi activity, that chain extension resulted in decreased activity in the pair of benzodioxole-containing hybrids (from **4AY3** to **4AY4**), while the pair of catechol-containing hybrids (from **4AY5** to **4AY6**) showed their activity slightly increasing, thus keeping the same trend observed for AChEi.

A final further comparison can still be made between the activities of the pairs of compounds (**4AY4** and **4AY6**) and (**4AY3** and **AY5**), each pair including, respectively, a catechol or a 1,3-benzodioxole group in the aromatic acid moiety, and the same chain length. Regarding AChEi, the catechol-containing hybrid (**4AY6**) shows higher activity than the benzodioxole derivative (**4AY4**), while an opposite effect is noticed in the corresponding pair of shorter size hybrids (**4AY5** and **4AY3**). However, concerning BChEi, both catechol hybrids present higher activity than the corresponding benzodioxole analogues, though with much closer IC_50_ values.

Overall, the hybridization with bigger size aromatic units with antioxidant activity, as the derivatives of syringic acid (**4AY1**, **4BY1**, **4CY1**) and Trolox (**4AY2**, **4BY2**) generally somehow compromised ChE’s inhibition, with higher impact in AChE (with a shorter active site cavity) than in BChE, making also their activity more dependent on the size of the linker between the aromatic moieties. Regarding the hybrids with caffeic acid derivatives (**4AYi**, i = 3–6), the presence of free catechol groups, in comparison with the corresponding benzodioxole groups, generally leads to good ChEi activity, though also better BChEi than AChEi, and, in most cases, with dependence on the linker size.

### 4.4. Inhibition of Self Aβ_1–42_ Aggregation

The inhibition of amyloid peptide aggregation, which ultimately can lead to the formation of amyloid plaques, is of paramount importance in treating AD. Therefore, we have assessed all the hybrids for their capacity to inhibit Aβ_1–42_ aggregation, employing a reported method based on the fluorescence emission of thioflavin T [33,34].

A brief analysis of structure–activity relationships, namely concerning the length of the linker between the RIV and the aromatic acid moieties, shows that the impact of adding one methylene group depends on the last moiety. In fact, this structural modification increases the %Aβ self-aggregation inhibitory activity nearly threefold in the syringic acid derivatives (**4AY1** and **4BY1**), while in the racemic mixtures of the Trolox derivatives (**4AY2** to obtain **4BY2**), similar good values were obtained. And it is reduced *ca* 36% for the caffeic acid derivatives (**4AY3** and **4BY3**). Moreover, the extension of the conjugation with one alkene group in caffeic acid derivatives (**4AY5** to get **4AY6**) produces high inhibitory ability, similar to curcumin. Furthermore, when comparing the results obtained for **4AY3** and **4AY5**, which structure difference lies just in the change of a 1,3-benzodioxole to a catechol group in the aromatic carboxylic acid moiety, there is about twofold enhancement in the inhibitory capacity. Since compounds **4AY4** and **4CY1** have a poorer solubility in an aqueous medium, a lower concentration of inhibitor was used (20 μM). So, the corresponding results also reflect this difference in concentration conditions, which makes it more difficult to compare these two compounds. However, based on the differences in the experimental conditions, it can be suggested that the relationship found for the pair **4AY3**/**4AY5** is followed in the pair **4AY4**/**4AY6**. In further studies, it could be interesting to test the effect of both chiral compounds (**4AY2** and **4BY2**, with similar activity) as inhibitors of Aβ aggregation, to search for possible synergistic or antagonistic effects, although herein it was decided to follow a one-drug multitarget strategy.

Overall, the obtained results indicated that, among this set of hybrids, the inclusion of catechol groups has a major contribution to the enhancement of their Aβ aggregation inhibitory activity, otherwise according to previously found in natural polyphenolic compounds [66].

### 4.5. Molecular-Docking Studies

The primary pathophysiological strategy underlying the development of currently FDA-approved drugs for AD involves compensating for the decline in the neurotransmitter acetylcholine (ACh). This cholinergic deficit arises from the progressive degeneration of cholinergic neurons, a process closely associated with the activity of the serine hydrolases acetylcholinesterase (AChE) and butyrylcholinesterase (BChE). Beyond their canonical roles in ACh hydrolysis, both enzymes have also been implicated in the pathogenesis of AD, particularly in amyloid-β (Aβ) aggregation, neurotoxicity, and plaque maturation. While AChE exhibits high substrate specificity for ACh, BChE possesses broader substrate promiscuity, enabling it to hydrolyze a wider range of esters and neuroactive peptides [67].

Thus, it is essential to recognize the structural differences between *h*AChE and *h*BChE to guide the rational design of effective and selective inhibitors. Although the two human enzymes share approximately 65% of amino acid sequence identity, they exhibit distinct structural features. Both possess a catalytic active site (CAS) located at the base of a deep, narrow, and predominantly hydrophobic gorge (ca 20 Ẳ in depth), as well as a peripheral anionic site (PAS) positioned near the entrance of the gorge [68,69]. However, amino acid composition and gorge architecture differences influence substrate specificity, ligand binding, and inhibitor selectivity between the two enzymes. These structural differences are mainly attributed to the number and type of aromatic residues lining the active-site gorge, which influence the overall shape and volume of the gorge. Specifically, the *h*BChE gorge is broader and less constrained than that of *h*AChE, facilitating the accommodation of bulkier ligands and providing easier access to the catalytic site [70,71]. Despite these differences, the active sites of both enzymes share common structural features, including a conserved catalytic triad composed of serine, histidine, and glutamate residues, as well as an acyl-binding pocket and a choline-binding site, which are critical for substrate recognition and catalytic activity.

The performed molecular-docking studies do not allow for establishing a clear correlation between the docking orientation of the hybrids in *h*AChE and their in vitro inhibitory activity. In fact, most assayed hybrids adopted a binding orientation comparable to the most potent ones (**4AY4** and **4AY6**), suggesting that, in addition to the differences between *ee*AChE and *h*AChE, orientation alone does not account for the observed differences in potency.

An analysis of the structural modifications within the hybrids revealed a notable impact on their inhibitory activity against *ee*AChE. Specifically, adding an extra alkene group was associated with a marked enhancement in activity. For example, compound **4AY4** (IC_50_ = 14.55 µM) demonstrated significantly greater potency than its structurally simpler analogue **4AY3** (IC_50_ = 50.5 µM), and a similar trend was observed between **4AY6** (IC_50_ = 5 µM) and **4AY5** (IC_50_ = 110 µM). This structural modification appears to influence the orientation and accommodation of the compounds within the enzyme’s binding site, potentially enhancing key interactions. Concerning the racemic mixtures, since the enantiomers of **4BY2** appear to have a higher degree of superimposition compared to those of **4AY2**, the enantiomers of **4BY2** may exhibit more similar biological activity than those of **4AY2**.

Although no direct correlation was observed between the docking scores and the experimental AChE inhibitory activity, some trends were consistent with the in vitro results. Compounds **4AY1**, **4AY5**, and **4CY1**, which displayed lower experimental inhibitory activity, also exhibited significantly less favorable docking scores than donepezil. Interestingly, the enantiomers **R4BY2** and **S4BY2** achieved the highest docking scores, aligning with the relatively strong inhibitory activity observed for **4BY2**, which ranked third among the tested compounds. It is well-recognized that achieving a perfect correlation between docking scores and biological activity is challenging due to limitations in scoring functions, since scoring functions often fail to fully capture the complexity of receptor–ligand binding affinity and dynamics [72]. This underlies the importance of examining the qualitative nature of molecular interactions within the enzyme’s active site to complement the docking scores (see Section 3.5).

As observed for *h*AChE, no clear correlation could be established between the spatial orientation of the RIV moiety and the compounds’ inhibitory potency toward BChE. Structural variations within the hybrid molecules influenced their conformations within the active site but did not consistently predict biological activity. For the chiral compounds **4AY2** and **4BY2**, significant changes in binding positioning were observed between the R and S isomers and those of potent inhibitors, the findings suggesting that, for *h*BChE, one enantiomer may exhibit superior inhibitory activity compared to its stereoisomer.

In conclusion, although the docking studies did not reveal a consistent relationship between ligand orientation in the active site and inhibitory potency determined in vitro, they provided valuable insights into structural-binding determinants and highlighted potential key residues involved in ChE inhibition.

### 4.6. Relevance of Findings to Neurodegeneration and Therapeutic Potential

Despite the multifactorial nature of Alzheimer’s disease (AD), two key pathological features remain central to its progression: the accumulation of senile plaques formed by amyloid-β (Aβ) aggregates—particularly the neurotoxic Aβ_1–42_ isoform—and increased oxidative stress through the production of reactive oxygen species (ROS) [73,74]. In line with this, our experiments demonstrated a significant decrease in cell viability upon exposure to Aβ_1–42_ and to the oxidative stress induced by iron/ascorbate treatment. The cell assays herein performed (compound administration prior to Aβ_1–42_ exposure) were able to assess the potential neuroprotective effects of the compounds by mimicking a prophylactic or early intervention scenario, which is commonly used in in vitro models to understand the mechanism of action and screen neuroprotective agents. Alternatively, studies including the administration of the compounds after Aβ_1–42_ exposure could have been performed at different times (short and long term protection), as well as repetitive treatments could be analyzed if only short term protection was verified, thus modeling a therapeutic scenario where amyloid aggregation would be already present.

The herein obtained results revealed that only in the context of oxidative stress did compounds **4AY2**, **4AY4**, and **4BY2** show protective effects. Specifically, these compounds significantly improved cell viability following iron/ascorbate exposure, restoring levels close to those observed in untreated controls. In contrast, none of the tested compounds were able to prevent the cytotoxicity induced by Aβ_1–42_.

Recent evidence suggests that mitochondrial dysfunction, often exacerbated by environmental factors, plays a pivotal role in the pathogenesis of AD [75,76]. Therefore, the observed protective effects of selected RIV-derived compounds against oxidative damage highlight their potential to modulate mitochondrial or redox homeostasis, though further studies are required to elucidate their mechanisms of action and their relevance to Aβ-associated toxicity.

### 4.7. Prediction of Pharmacokinetic and Physicochemical Properties

Some of the pharmacokinetic parameters of the herein developed compounds (in Table 2) were calculated to anticipate their potential as therapeutic agents.

Compared with the rivastigmine drug, all the hybrids exhibit higher PSA values, which has a significant role in drug design because polarity can restrict entry into the human body, though the calculated values are still within an acceptable range. The *c*log P_o/w_ values have also increased with the RIV hybridization, indicating that the hybrids are more lipophilic and, thus, with enhanced ability to cross cell membranes, compared to rivastigmine. Regarding their interaction with human serum albumin (HSA), the predicted values for all the rivastigmine hybrids are higher than for rivastigmine, showing that their availability to bind or interact with the target protein should be more reduced, since only the unbound fraction can leave the bloodstream, reach its site of action, and exert a pharmacological effect. On the other hand, high HSA binding can decrease the compound’s clearance rate.

Concerning log BB values, they are lower than those of rivastigmine, while for the Caco-2 and MDCK values, the hybrids showed varied predicted permeabilities. For the permeability capacity, all the values are favorable, except for compounds **4AY5** and **4AY6**, which yield moderate–good values. In fact, some compounds exhibit higher values than rivastigmine, while others present lower ones, highlighting variability in their ability to penetrate the intestinal barrier of enteric cells and cross the brain–blood barrier (BBB).

Finally, noteworthy is the fact that none of the compounds (even the chiral ones) violate Lipinski’s Rule of Five, suggesting they can be considered as potential oral drug candidates.

## 5. Conclusions

A series of novel compounds, generated through the hybridization of the cholinesterase inhibitor rivastigmine with several antioxidant aromatic moieties, was developed and evaluated for multiple biological activities with potential relevance to AD therapy. In addition to their expected activity against both AChE and BChE, conferred by the RIV core, some of these hybrids also demonstrated significant antioxidant properties and ability to inhibit the amyloid β-peptide (Aβ) self-aggregation. The biological activity profiles of these compounds were influenced by the type of the antioxidant motifs, the number of free phenolic hydroxyl groups, and the type and size of the linker between the two main moieties. As anticipated, the most potent antioxidant (radical-scavenging) activity was observed in the Trolox-based hybrids (**4AY2**, **4BY2**) and those containing free catechol groups (**4AY5**, **4AY6**). Regarding the cholinesterase inhibition, hybrids incorporating larger antioxidant or pro-antioxidant aromatic unities exhibited, somehow, a reduced activity compared to the parent rivastigmine compound, particularly against AChE, which has a narrower active site. Concerning the inhibition of BChE, among the tested compounds, the syringic acid derivatives and the catechol-based hybrids showed, respectivelly, the lowest and higher activities, while 4BY3 exhibited the most potent inhibitory activity, similarly to rivastigmine. All hybrids could inhibit Aβ self-aggregation but, generally, the catechol-containing compounds evidenced higher activity. Compounds **4AY2**, **4AY4**, and **4BY2** showed protective effect in cells, with potential to modulate mitochondrial or redox homeostasis, but none of the compounds assayed were able to avoid the cytotoxicity induced by Aβ_1–42_. Molecular docking studies revealed key structural features and binding interactions of RIV-based hybrids with both AChE and BChE, highlighting recurrent interactions with residues such as TRP86, TYR337, HIS447, and SER198. Although no general consistent correlation was found between docking scores and experimental inhibitory potency, certain structural modifications, particularly incorporating an alkene group, were associated with improved activity, likely due to enhanced accommodation within the enzyme binding site. Differences in binding orientation between enantiomers were also observed, suggesting possible stereoselectivity in BChE inhibition. These findings underscore the importance of qualitative interaction analysis in complementing docking scores and elucidating structure-activity relationships. Together, these results and favorable predicted pharmacokinetic profiles support the potential of some RIV-based hybrids as promising hit compounds for further investigation in the context of AD, particularly as multifunctional agents targeting multiple pathological features of the disease. 

## Figures and Tables

**Table 1:** Biological activity for the studied set of RIV hybrids as well as reference compounds.

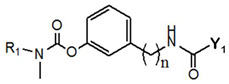									
Compound	R_1_	n	% AA ^a^	AA ^a^ EC_50_ (μM)	AChE Inhib ^b^ IC_50_ (μM)	BChE Inhib ^b^ IC_50_ (μM)	SI ^c^	% Aβ_42_ Self-Agg Inhib ^d^	
**4AY1**	Et	0	<50	-	>200	31 ± 2	-	20 ± 2	
**4AY2**	Et	0	95 ± 3	18.1 ± 0.4	69 ± 7	36 ± 3	1.9	45 ± 4	
**4AY3**	Et	0	<50	-	50.5 ± 0.1	3.1 ± 0.4	16.3	45 ± 4	
**4AY4**	Et	0	<50	-	14.51 ± 0.04	15.2 ± 0.4	0.9	23 ± 3 ^e^	
**4AY5**	Et	0	96 ± 2	15.7 ± 0.4	110 ± 5	7.2 ± 0.3	15.3	82 ± 5	
**4AY6**	Et	0	93.1 ± 0.8	28.0 ± 0.4	5 ± 1	5.7 ± 0.6	0.9	75 ± 7	
**4BY1**	Et	1	<50	-	>200	75.0 ± 0.6	-	58 ± 6	
**4BY2**	Et	1	93.9 ± 0.4	20.0 ± 0.8	32 ± 2	3.7 ± 0.1	8.6	47 ± 3	
**4BY3**	Et	1	<50	-	91 ± 6	0.9 ± 0.2	101	29 ± 3	
**4CY1**	Me	0	<50	-	>200	68 ± 7	-	31 ± 3 ^e^	
Trolox	-	-	-	13.8 ± 0.2	-	-	-	-	
Rivastigmine ^f^	-	-	-	-	32 ± 1	0.39 ± 0.09	82.3	-	
Curcumin	-	-	-	-	-	-	-	77 ± 1	
One-way ANOVA	-	-						C_L_ = 20 µM ^e^	C_L_ = 40 µM
Compounds			4AY(2,5,6), 4BY2	4AY(2,5,6), 4BY2, Trolox	4AY(2–6), 4BY(2,3), rivastigmine	4AY(1–6), 4BY(1–3), 4CY1, rivastigmine		4AY4, 4CY1	4AY(1–3,5,6), 4BY(1–3), curcumin
Numerator			4.134	91.01	2528.7	1468.4		120.12	1901.6
Denominator			4.108	0.274	33.22	10.40		15.01	25.79
F value			F(3,8) = 1.006	F(4,10) = 331.6	F(7,8) = 76.13	F(10,11) = 141.2		F(1,6) = 8.002	F(8,27) = 73.74
*p*-value			0.439	<0.001	<0.001	<0.001		0.030	<0.001

^a^ Mean values of three independent experiments (DPPH method); ^b^ mean values of two independent experiments ± SD, performed with *ee*AChE and *eq*BChE; ^c^ selectivity index: SI = IC_50_ (AChE)/IC_50_ (BChE); ^d^ mean values of four independent measurements in duplicate for inhibition of Aβ_42_ aggregation (C_inhibitor_ = 40 μM) (ThT fluorescence method); ^e^ ThT fluorescence assays (C_inhibitor_ = 20 μM); ^f^ Ref. [23]. For each type of assay, one-way ANOVA was used, showing that the results are statistically different at a 95% confidence level (*p* < 0.05), except for % AA assays (*p* > 0.05). Post hoc tests were carried out for AA EC_50_, IC_50_ (AChE), IC_50_ (BChE), and % Aβ Self-Agg Inhib (assays for which the null hypothesis was rejected), comparing each compound to the respective reference compound: for each assay, all the compounds are significantly different from the control, except **4BY2** relative to rivastigmine for IC_50_ (AChE), **4BY3** relative to rivastigmine for IC_50_ (BChE) and **4AY5** and **4AY6** relative to curcumin for % Aβ Self-Agg Inhib (see Table S1).

**Table 2:** Predicted pharmacokinetic properties of the RIV hybrids, as well as the parent Rivastigmine drug found with QikProp v.2.5 [57].

Compound	MW ^a^	PSA ^b^	*c*log P_o/w_ ^c^	log *K* (HSA) ^d^	log BB ^e^	Caco-2 Permeab. ^f^	MDCK Permeab. ^g^	% Oral Absorption ^h^
**4AY1**	374.393	99.757	3.079	0.043	−0.783	1437	732	91
**4AY2**	426.511	88.438/ 92.26	4.479/ 4.565	0.759/ 0.784	−0.66/ −0.699	1449/ 1420	739/ 723	86/ 87
**4AY3**	368.388	91.183	3.444	0.143	−0.615	1733	896	100
**4AY4**	394.426	91.452	4.123	0.327	−0.797	1699	877	100
**4AY5**	356.377	115.137	2.512	0.032	−1.953	175	75	72
**4AY6**	382.415	115.078	3.143	0.187	−2.183	170	73	73
**4BY1**	388.419	102.759	3.19	0.129	−1.153	793	385	83
**4BY2**	440.538	79.002/ 94.477	3.882/ 3.979	0.338/ 0.507	−0.189/ −0.895	2193/ 513	1987/ 476	94/ 81
**4BY3**	382.415	94.46	3.627	0.252	−0.949	1016	503	91
**4CY1**	360.366	100.414	2.697	−0.039	−0.845	1122	561	88
Rivastigmine	250.34	38.483	2.448	−0.133	0.475	1381	776	100

^a^ acceptable range from 130 to 725; ^b^ acceptable range from 7 to 200; ^c^ acceptable range from −2.0 to 6.5; ^d^ acceptable range from −1.5 to +1.5; ^e^ acceptable range from −3.0 to +1.2; ^f^ values of 25 are considered poor and >500 are considered great; ^g^ with values <25 being poor and >500 being great; ^h^ <25% is considered poor, and >80% is considered high.

## Data Availability

Data are contained in this article and Supplementary Materials.

## Supplementary Materials

The following supporting information can be downloaded at https://www.mdpi.com/article/10.3390/antiox14080921/s1.

## References

[1] Cummings J., Zhou Y., Lee G., Zhong K., Fonseca J., Cheng F. (2024). Alzheimer’s disease drug development pipeline: 2024. Alzheimer’s Dement..

[2] Bhatt J., Commas-Herrera A., D’Amico F., Farina N., Wong J., Orange J., Gaber S., Knapp M., Salcher-Konrad M., Stevens M. (2019). The World Alzheimer Report 2019: Attitudes to Dementia.

[3] van der Kant R., Goldstein L.S.B., Ossenkoppele R. (2020). Amyloid-β-independent regulators of tau pathology in Alzheimer disease. Nat. Rev. Neurosc..

[4] Scarpini E., Schelterns P., Feldman H. (2003). Treatment of Alzheimer’s disease: Current status and new perspectives. Lancet Neurol..

[5] Greenough M.A., Camakaris J., Bush A.I. (2013). Metal dyshomeostasis and oxidative stress in Alzheimer’s disease. Neurochem. Int..

[6] Cummings J., Lee G., Nahed P., Kambar M.E.Z.N., Zhong K., Fonseca J., Taghva K. (2022). Alzheimer’s disease drug development pipeline: 2022. Alzheimer’s Dement..

[7] Zemek F., Drtinova L., Nepovimova E., Sepsova V., Korabecny J., Klimes J., Kuca K. (2014). Outcomes of Alzheimer’s disease therapy with acetylcholinesterase inhibitors and memantine. Expert. Opin. Drug Saf..

[8] Cummings J.L., Morstorf T., Zhong K. (2014). Alzheimer’s disease drug-development pipeline: Few candidates, frequent failures. Alzheimer’s Res. Ther..

[9] Calhoun A., King C., Khoury R., Grossberg G.T. (2018). An evaluation of memantine ER/donepezil for the treatment of Alzheimer’s disease. Expert. Opin. Pharmacother..

[10] Sevigny J., Chiao P., Bussière T., Weinreb P.H., Williams L., Maier M., Dunstan R., Salloway S., Chen T., Ling Y. (2016). The antibody aducanumab reduces Aβ plaques in Alzheimer’s disease. Nature.

[11] van Dyck C.H., Swanson C.J., Aisen P., Bateman R.J., Chen C., Gee M., Kanekiyo M., Li D., Reyderman L., Cohen S. (2023). Lecanemab in early Alzheimer’s disease. N. Engl. J. Med..

[12] Pardo-Moreno T., Gonzalez-Acedo A., Rivas-Dominguez A., Garcia-Morales V., Garcia-Cozar F.J., Ramos-Rodriguez J.J., Melguizo-Rodriguez L. (2022). Therapeutic approach to Alzheimer’s disease: Current treatments and new perspectives. Pharmaceutics.

[13] Albertini C., Salerno A., Pinheiro P.S.M., Bolognesi M.L. (2021). From combinations to multitarget-directed ligands: A continuum in Alzheimer’s disease polypharmacology. Med. Res. Rev..

[14] Oset-Gasque M.J., Marco-Contelles J. (2018). Alzheimer’s disease, the “One-Molecule, One-Target” paradigm, and the multitarget directed ligand approach. ACS Chem. Neurosci..

[15] Santos M.A., Chand K., Chaves S. (2016). Recent progress in repositioning Alzheimer’s disease drugs based on a multitarget strategy. Future Med. Chem..

[16] Sampietro A., Perez-Areales F.J., Martinez P., Arce E.M., Galdeano C., Munoz-Torrero D. (2022). Unveiling the multitarget anti-Alzheimer drug discovery landscape: A bibliometric analysis. Pharmaceuticals.

[17] Rossi M., Freschi M., Nascente L.C., Salerno A., Teixeira S.M.V., Nachon F., Chantegreil F., Soukup O., Prchal L., Malaguti M. (2021). Sustainable drug discovery of multi-target-directed ligands for Alzheimer’s disease. J. Med. Chem..

[18] Bautista-Aguilera Ó.M., Hagenow S., Palomino-Antolin A., Farré-Alins V., Ismaili L., Joffrin P.-L., Jimeno M.L., Soukup O., Janočková J., Kalinowsky L. (2017). Multitarget-directed ligands combining cholinesterase and monoamine oxidase inhibition with histamine H_3_R antagonism for neurodegenerative diseases. Angew. Chem. Int. Ed. Engl..

[19] Hiremathad A., Keri R.S., Esteves A.R., Cardoso S.M., Chaves S., Santos M.A. (2018). Novel Tacrine-hydroxyphenylbenzimidazole hybrids as potential multitarget drug candidates for Alzheimer’s disease. Eur. J. Med. Chem..

[20] Chaves S., Várnagy K., Santos M.A. (2021). Recent multi-target approaches on the development of anti-Alzheimer’s agents integrating metal chelation activity. Curr. Med. Chem..

[21] Lane R.M., Potkin S.G., Enz A. (2006). Targeting acetylcholinesterase and butyrylcholinesterase in dementia. Int. J. Neuropsychopharmacol..

[22] Liu Y., Ma C., Li Y., Li M., Cui T., Zhao X., Li Z., Jia H., Wang H., Xiu X. (2024). Design, synthesis and biological evaluation of carbamate derivatives incorporating multifunctional carrier scaffolds as pseudo-irreversible cholinesterase inhibitors for the treatment of Alzheimer’s disease. Eur. J. Med. Chem..

[23] Vicente-Zurdo D., Rosales-Conrado N., León-González M.E., Brunetti L., Piemontese L., Pereira-Santos A.R., Cardoso S.M., Madrid Y., Chaves S., Santos M.A. (2022). Novel rivastigmine derivatives as promising multi-target compounds for potential treatment of Alzheimer’s disease. Biomedicines.

[24] Bon L., Bana’s A., Dias I., Melo-Marques I., Cardoso S.M., Chaves S., Santos M.A. (2024). New multitarget rivastigmine–indole hybrids as potential drug candidates for Alzheimer’s disease. Pharmaceutics.

[25] Calsolaro V., Edison P. (2016). Neuroinflammation in Alzheimer’s disease: Current evidence and future directions. Alzheimer’s Dement..

[26] Kamaljeet, Singh S., Gupta G.D., Aran K.R. (2024). Emerging role of antioxidants in Alzheimer’s disease: Insight into physiological, pathological mechanisms and management. Pharmaceut. Sci. Adv..

[27] Mezeiova E., Spilovska K., Nepovimova E., Gorecki L., Soukup O., Dolezal R., Malinak D., Janockova J., Jun D., Kuca K. (2018). Profiling donepezil template into multipotent hybrids with antioxidant properties. J. Enz. Inhib. Med. Chem..

[28] Cai P., Fang S.-Q., Yang X.-L., Wu J.-J., Liu Q.-H., Hong H., Wang X.-B., Kong L.-Y. (2017). Rational design and multibiological profiling of novel donepezil−trolox hybrids against Alzheimer’s disease, with cholinergic, antioxidant, neuroprotective, and cognition enhancing properties. ACS Chem. Neurosci..

[29] Cao Y., Zhang L., Sun S., Yi Z., Jiang X., Jia D. (2016). Neuroprotective effects of syringic acid against OGD/R-induced injury in cultured hippocampal neuronal cells. Int. J. Mol. Med..

[30] Quintanova C., Keri R.S., Marques S.M., Fernandes M.G., Cardoso S.M., Serralheiro M.L., Santos M.A. (2015). Design, Synthesis and bioevaluation of tacrine-cinnamate based hybrids as potential bifunctional anti-Alzheimer drug candidates. MedChemComm.

[31] Chavarria D., Cagide F., Pinto M., Gomes L.R., Low J.N., Borges F. (2019). Development of piperic acid-based monoamine oxidase inhibitors: Synthesis, structural characterization and biological evaluation. J. Mol. Struct..

[32] Armarego W.L.F., Perrin D.D. (1999). Purification of Laboratory Chemicals.

[33] Bartolini M., Bertucci C., Bolognesi M.L., Cavalli A., Melchiorre C., Andrisano V. (2007). Insight into the kinetic of amyloid beta (1-42) peptide self-aggregation: Elucidation of inhibitors’ mechanism of action. ChemBioChem.

[34] Chao X., He X., Yang Y., Zhou X., Jin M., Liu S., Cheng Z., Liu P., Wang Y., Yu J. (2012). Design, synthesis and pharmacological evaluation of novel tacrine-caffeic acid hybrids as multi-targeted compounds against Alzheimer’s disease. Bioorg. Med. Chem. Lett..

[35] (2025). Crystallographic Structure Factors and Electron Density, Resolution and R-Value, RCSB PDB-101. https://pdb101.rcsb.org/learn/guide-to-understanding-pdb-data/crystallographic-data.

[36] Berman H.M., Battistuz T., Bhat T.N., Bluhm W.F., Bourne P.E., Burkhardt K., Feng Z., Gilliland G.L., Iype L., Jain S. (2000). The Protein Data Bank. Nucleic Acids Res..

[37] Cheung J., Rudolph M.J., Burshteyn F., Cassidy M.S., Gary E.N., Love J., Franklin M.C., Height J.J. (2012). Structures of human acetylcholinesterase in complex with pharmacologically important ligands. J. Med. Chem..

[38] Brus B., Košak U., Turk S., Pišlar A., Coquelle N., Kos J., Stojan J., Colletier J.-P., Gobec S. (2014). Discovery, biological evaluation, and crystal structure of a novel nanomolar selective butyrylcholinesterase inhibitor. J. Med. Chem..

[39] Košak U., Knez D., Coquelle N., Brus B., Pišlar A., Nachon F., Brazzolotto X., Kos J., Colletier J., Gobe S. (2017). N-propargylpiperidines with naphthalene-2-carboxamide or naphthalene-2-sulfonamide moieties: Potential multifunctional anti-Alzheimer’s agents. Bioorg. Med. Chem..

[40] Viayna E., Coquelle N., Cieslikiewicz-Bouet M., Cisternas P., Oliva C.A., Sánchez-López E., Ettcheto M., Bartolini M., De Simone A., Ricchini M. (2021). Discovery of a potent dual inhibitor of acetylcholinesterase and butyrylcholinesterase with antioxidant activity that alleviates Alzheimer-like pathology in old APP/PS1 mice. J. Med. Chem..

[41] (2025). Molecular Operating Environment (MOE) 2024.0601.

[42] Fadlan A., Nusantoro Y.R. (2021). The effect of energy minimization on the molecular docking of acetone-based oxindole derivatives. J. Kim. Dan. Pendidik. Kim..

[43] (2012). Maestro.

[44] Che X., Liu Q., Zhang L. (2023). An accurate and universal protein-small molecule batch docking solution using Autodock Vina. Results Eng..

[45] Hoffmann R. (1963). An extended Hückel theory. I. Hydrocarbons. J. Chem. Phys..

[46] Jones G., Willett P., Glen R.C., Leach A.R., Taylor R. (1997). Development and validation of a genetic algorithm for flexible docking. J. Mol. Biol..

[47] Gehlhaar D.K., Verkhivker G.M., Rejto P.A., Sherman C.J., Fogel D.B., Fogel L.J., Freer S.T. (1995). Molecular recognition of the inhibitor AG-1343 by HIV-1 protease: Conformationally flexible docking by evolutionary programming. Chem. Biol..

[48] Eldridge M.D., Murray C.W., Auton T.R., Paolini G.V., Mee R.P. (1997). Empirical scoring functions: I. The development of a fast empirical scoring function to estimate the binding affinity of ligands in receptor complexes. J. Comput. Aided. Mol. Des..

[49] Mooij W.T.M., Verdonk M.L. (2005). General and targeted statistical potentials for protein-ligand interactions. Proteins Struct. Funct. Genet..

[50] McNutt A.T., Francoeur P., Aggarwal R., Masuda T., Meli R., Ragoza M., Sunseri J., Koes D.R. (2021). GNINA 1.0: Molecular docking with deep learning. J. Cheminform..

[51] Koes D.R., Baumgartner M.P., Camacho C.J. (2013). Lessons learned in empirical scoring with smina from the CSAR 2011 benchmarking exercise. J. Chem. Inf. Model..

[52] Friesner R.A., Banks J.L., Murphy R.B., Halgren T.A., Klicic J.J., Mainz D.T., Repasky M.P., Knoll E.H., Shelley M., Perry J.K. (2004). Glide: A New Approach for Rapid, Accurate Docking and Scoring. 1. Method and Assessment of Docking Accuracy. J. Med. Chem..

[53] Friesner R.A., Murphy R.B., Repasky M.P., Frye L.L., Greenwood J.R., Halgren T.A., Sanschagrin P.C., Mainz D.T. (2006). Extra precision glide: Docking and scoring incorporating a model of hydrophobic enclosure for protein-ligand complexes. J. Med. Chem..

[54] Neudert G., Klebe G. (2011). fconv: Format conversion, manipulation and feature computation of molecular data. Bioinformatics.

[55] Bouysset C., Fiorucci S. (2021). ProLIF: A library to encode molecular interactions as fingerprints. J. Cheminform..

[56] Mosmann T. (1983). Rapid colorimetric assay for cellular growth and survival: Application to proliferation and cytotoxicity assays. J. Immunol. Methods.

[57] (2005). QikProp.

[58] Salazar P.B., Dupuy F.G., Fiori M.C., Stanfield S.M., McCord J., Altenberg G.A., Minahk C.J. (2024). Nanodisc-associated acetylcholinesterase as a novel model system of physiological relevant membrane-bound cholinesterases. Inhibition by phenolic compounds. Biochem. Bioph. Acta—Biomembr..

[59] Bosak A., Gazic I., Vinkovic V., Kovarik Z. (2008). Stereoselective inhibition of human, mouse, and horse cholinesterases by bambuterol enantiomers. Chem. Biol. Interact..

[60] Xu Q., Zhang L., Xia G., Zhan D., Zhu J., Zang H. (2022). Synthesis and biological activity of trolox amide derivatives. Braz. J. Pharm. Sci..

[61] Chen J., Yang J., Ma L., Li J., Shahzad N., Kim C.K. (2020). Structure-antioxidant activity relationship of methoxy, phenolic hydroxyl, and carboxylic acid groups of phenolic acids. Sci. Rep..

[62] Chavarria D., Silva T., Martins D., Bravo J., Summavielle T., Garrido J., Borges F. (2015). Exploring cinnamic acid scaffold: Development of promising neuroprotective lipophilic antioxidants. MedChemComm.

[63] Kumar J., Shankar G., Kumar S., Thomas J., Singh N., Srikrishna S., Satija J., Krishnamurthy S., Modi G., Mishra S.K. (2023). Extraction, isolation, synthesis, and biological evaluation of novel piperic acid derivatives for the treatment of Alzheimer’s disease. Mol. Divers..

[64] Lee S.Y., Hwang I.Y., Jeong C.S. (2017). Protective effects of cinnamic acid derivatives on gastric lesion. Nat. Prod. Sci..

[65] Forero-Doria O., Guzmán L., Venturini W., Zapata-Gomez F., Duarte Y., Camargo-Ayala L., Echeverría C., Echeverría J. (2024). O-Alkyl derivatives of ferulic and syringic acid as lipophilic antioxidants: Effect of the length of the alkyl chain on the improvement of the thermo-oxidative stability of sunflower oil. RSC Adv..

[66] Pagano K., Tomaselli S., Molinari H., Ragona L. (2020). Natural compounds as inhibitors of Aβ peptide aggregation: Chemical requirements and molecular mechanisms. Front. Neurosci..

[67] Ballard C.G. (2002). Advances in the treatment of Alzheimer’s disease: Benefits of dual cholinesterase inhibition. Eur. Neurol..

[68] Harel M., Sussman J.L., Krejci E., Bon S., Chanal P., Massoulie J., Silman I. (1992). Conversion of acetylcholinesterase to butyrylcholinesterase: Modelling and mutagenesis. Proc. Natl. Acad. Sci. USA..

[69] Chatonnet A., Lockridge O. (1989). Comparison of butyrylcholinesterase and acetylcholinesterase. Biochem. J..

[70] Rosenberry T.L., Brazzolotto X., Macdonald I.R., Wandhammer M., Trovaslet-Leroy M., Darvesh S., Nachon F. (2017). Comparison of the binding of reversible inhibitors to human butyrylcholinesterase and acetylcholinesterase: A crystallographic, kinetic and calorimetric study. Molecules.

[71] Dighe S.N., Deora G.S., Mora E., Nachon F., Chan S., Parat M.O., Brazzolotto X., Ross B.P. (2016). Discovery and structure-activity relationships of a highly selective butyrylcholinesterase inhibitor by structure-based virtual screening. J. Med. Chem..

[72] Sliwoski G.R., Meiler J., Lowe E.W. (2014). Computational methods in drug discovery. Pharmacol. Rev..

[73] Silva D.F., Selfridge J.E., Lu J., Lezi E., Cardoso S.M., Swerdlow R.H. (2012). Mitochondrial abnormalities in Alzheimer’s disease: Possible targets for therapeutic intervention. Adv. Pharmacol..

[74] Candeias E., Pereira-Santos A.R., Empadinhas N., Cardoso S.M., Esteves A.R.F. (2024). The gut-brain axis in Alzheimer’s and Parkinson’s diseases: The catalytic role of mitochondria. J. Alzheimer’s Dis..

[75] Silva D.F., Candeias E., Esteves A.R., Magalhães J.D., Ferreira I.L., Nunes-Costa D., Rego A.C., Empadinhas N., Cardoso S.M. (2020). Microbial BMAA elicits mitochondrial dysfunction, innate immunity activation, and Alzheimer’s disease features in cortical neurons. J. Neuroinflammation.

[76] Silva D.F., Esteves A.R., Arduino D.M., Oliveira C.R., Cardoso S.M. (2011). Amyloid-β-induced mitochondrial dysfunction impairs the autophagic lysosomal pathway in a tubulin dependent pathway. J. Alzheimer’s Dis..

