# Supplementary material for: Rivastigmine Templates with Antioxidant Motifs—A Medicinal Chemist’s Toolbox Towards New Multipotent AD Drugs
Source: Antioxidants (Basel). 2025 Jul 28;14(8):921. doi: 10.3390/antiox14080921 (PMC12383012; doi:10.3390/antiox14080921)
Supplement: Supplementary file 1 [file antioxidants-14-00921-s001.zip › antioxidants-3672135-supplementary-7.30.pdf]

Supplementary Material

Rivastigmine Templates with Antioxidant Motifs - A Medicinal Chemist's Toolbox Towards New Multipotent AD Drugs

Inês Dias,[1] Marlène Emmanuel,[1] Paul Vogt,[1] Catarina Guerreiro-Oliveira,[2,3†] Inês Melo-Marques,[2,3†] Sandra M. Cardoso,[2,3,4] Rita C. Guedes,[5] Sílvia Chaves,[1*] M. Amélia Santos[1*]

[1]Centro de Química Estrutural, Institute of Molecular Sciences, Departamento de Engenharia Química, Instituto Superior Técnico, Universidade de Lisboa, Av. Rovisco Pais 1, 1049-001 Lisboa, Portugal
[2]CNC-UC, Center for Neuroscience and Cell Biology, Universidade de Coimbra, 3004-504 Coimbra, Portugal
[3]Centre for Innovative Biomedicine and Biotechnology, Universidade de Coimbra, 3004-504 Coimbra, Portugal
[4]FMUC, Faculdade de Medicina, Universidade de Coimbra, 3004-504 Coimbra, Portugal
[5]Research Institute for Medicines (iMed.ULisboa), Faculdade de Farmácia, Universidade de Lisboa, 1649-003 Lisboa, Portugal

1. Spectral data of the final synthesized compounds

(insets: molecular structure and simulated peak attributions (*chemdraw*)

1.1. (3-(4-Hydroxy-3,5-dimethoxybenzamido)phenyl ethyl(methyl)carbamate) (4AY1)

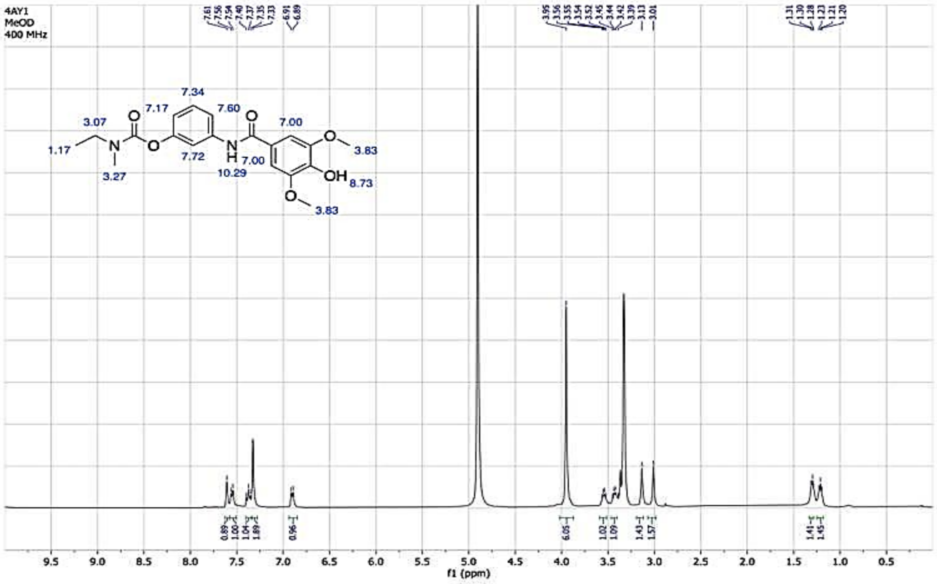

Figure S1. **4AY1** ^{1}H NMR spectrum.

1.2. 3-(6-Hydroxy-2,5,7,8-tetramethylchromane-2-carboxamido)phenylethyl(methyl)carbamate (4AY2)

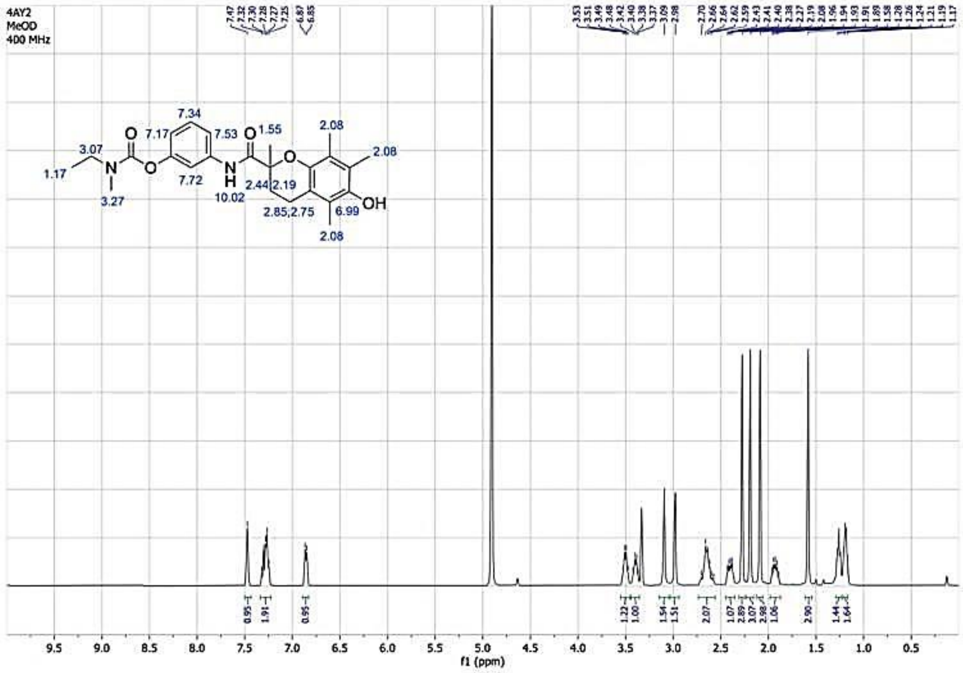

Figure S2. **4AY2** ^{1}H NMR spectrum.

1.3. (*E*)-3-(3-(benzo[*d*][1,3]dioxol-5-yl)acrylamido)phenyl ethyl(methyl)carbamate (4AY3)

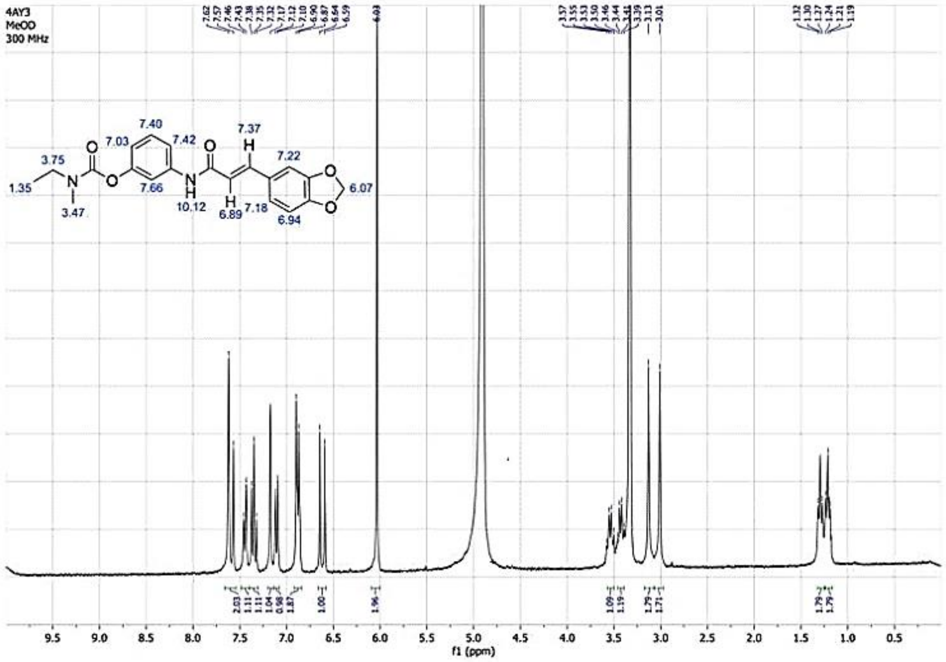

Figure S3. **4AY3** ^{1}H NMR spectrum.

1.4. 3-((2*E*,4*E*)-5-(benzo[*d*][1,3]dioxol-5-yl)penta-2,4-dienamido)phenylethyl(methyl)carbamate (4AY4)

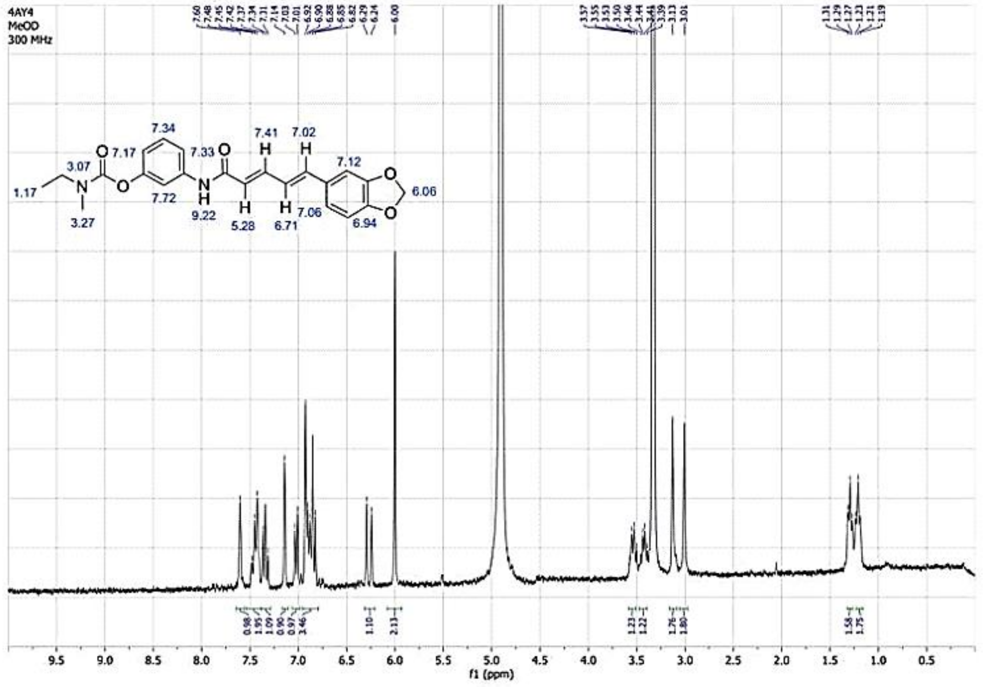

Figure S4. **4AY4** ^{1}H NMR spectrum.

1.5. (*E*)-3-(3-(3,4-dihydroxyphenyl)acrylamido)phenyl ethyl(methyl)carbamate (4AY5)

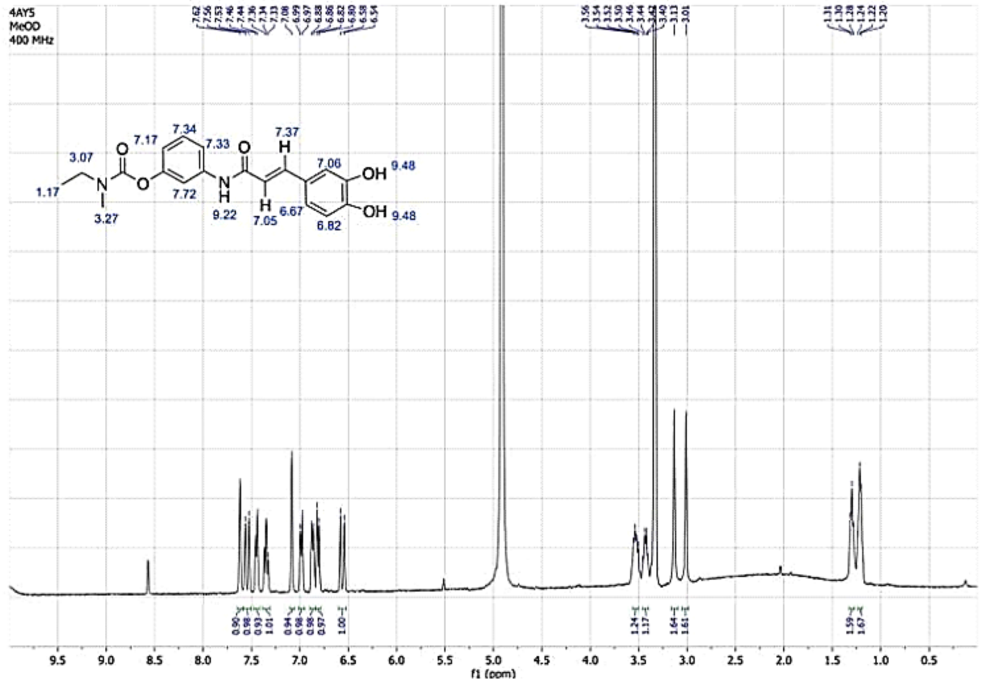

Figure S5. **4AY5** ^{1}H NMR spectrum.

1.6. 3-((2*E*,4*E*)-5-(3,4-Dihydroxyphenyl)penta-2,4-dienamido)phenyl ethyl(methyl)carbamate (4AY6)

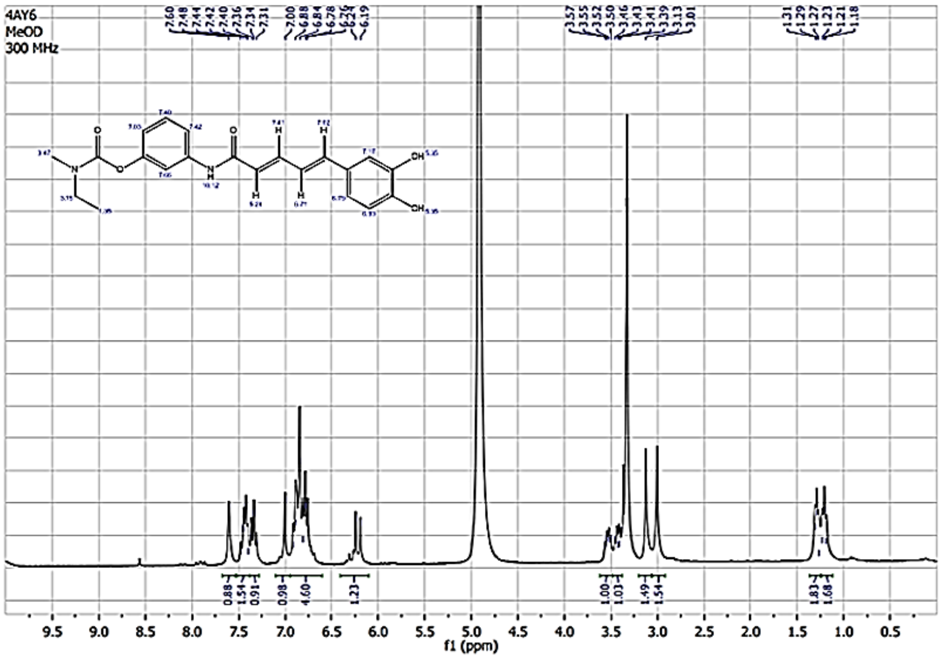

Figure S6. **4AY6** ^{1}H NMR spectrum.

1.7. 3-((4-Hydroxy-3,5-dimethoxybenzamido)methyl)phenyl ethyl(methyl)carbamate (4BY1)

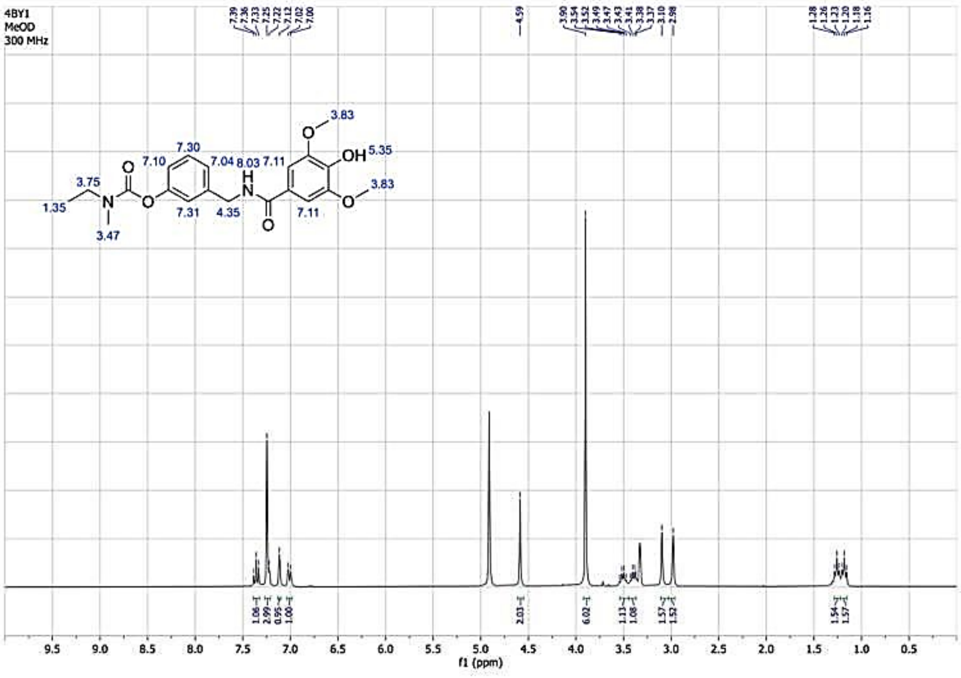

Figure S7. **4BY1** ^{1}H NMR spectrum.

1.8. 3-((6-Hydroxy-2,5,7,8-tetramethylchromane-2-carboxamido)methyl)phenyl ethyl(methyl)carbamate (4BY2)

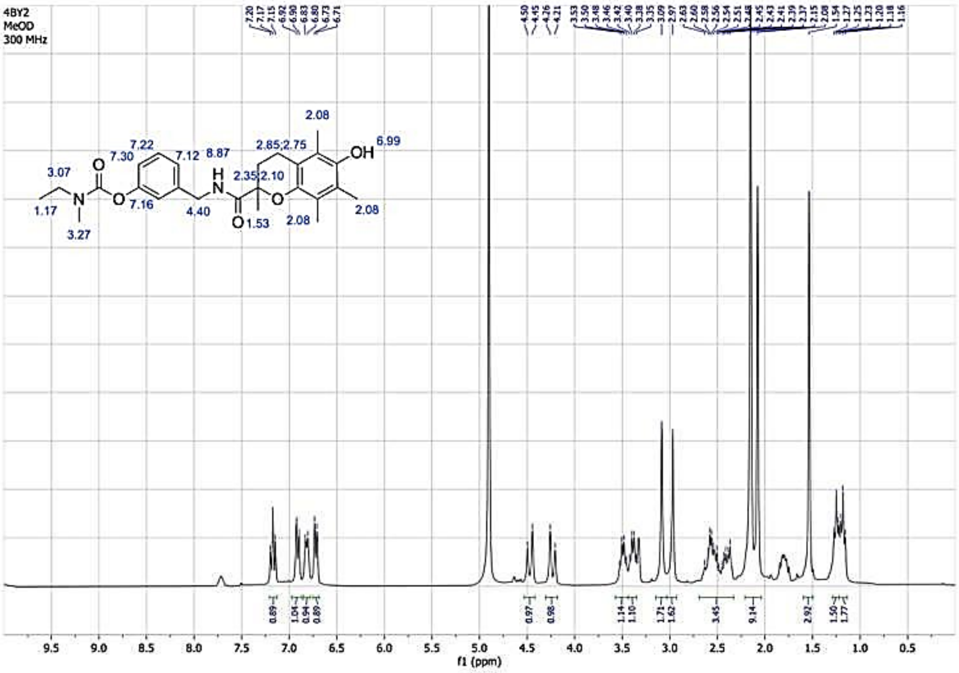

Figure S8. **4BY2** ^{1}H NMR spectrum.

1.9. (*E*)-3-((3-(benzo[*d*][1,3]dioxol-5-yl)acrylamido)methyl)phenyl ethyl(methyl)carbamate (4BY3)

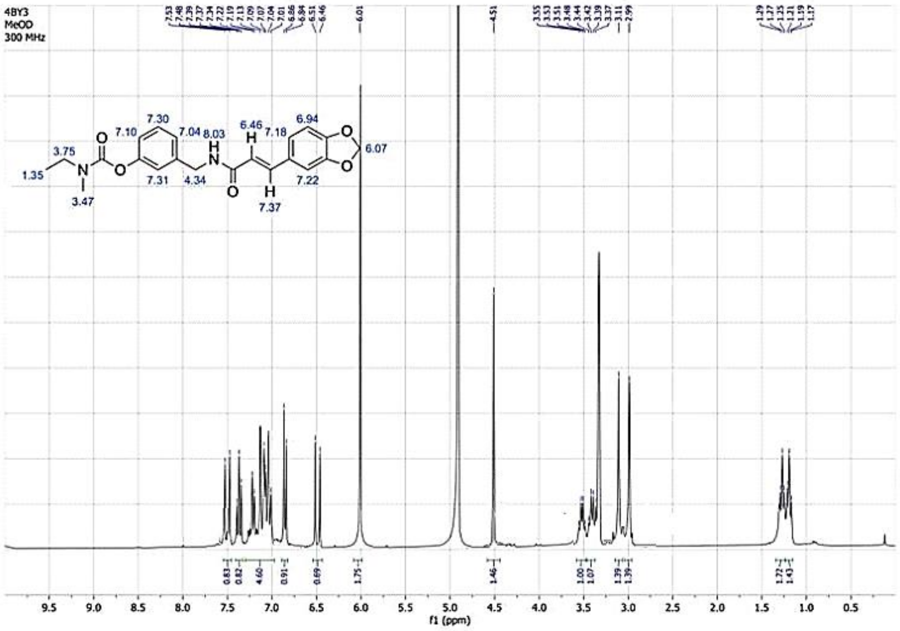

Figure S9. **4BY3** ^{1}H NMR spectrum.

1.10. 3-(4-Hydroxy-3,5-dimethoxybenzamido)phenyl dimethylcarbamate (4CY1)

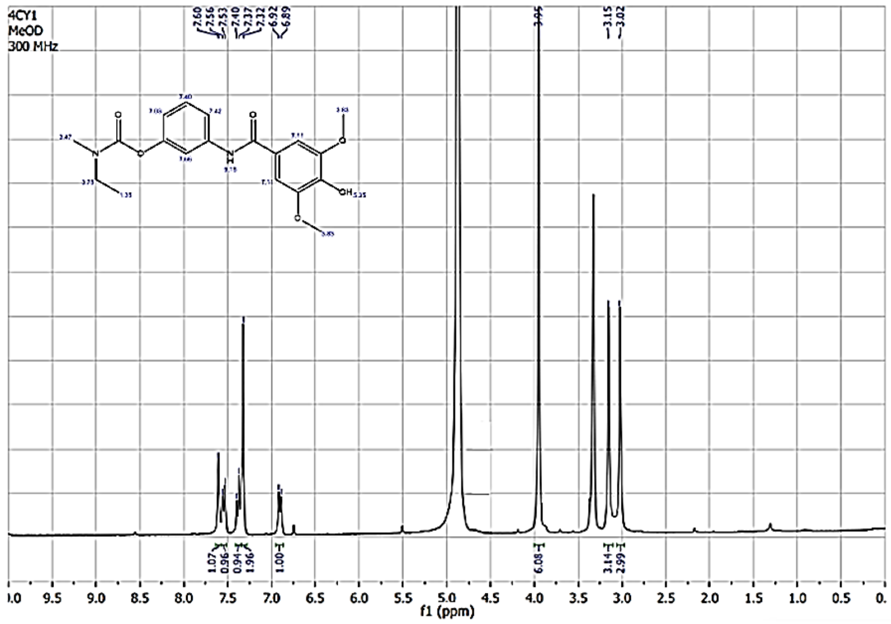

Figure S10. **4CY1** ^{1}H NMR spectrum.

2. Molecular docking figures

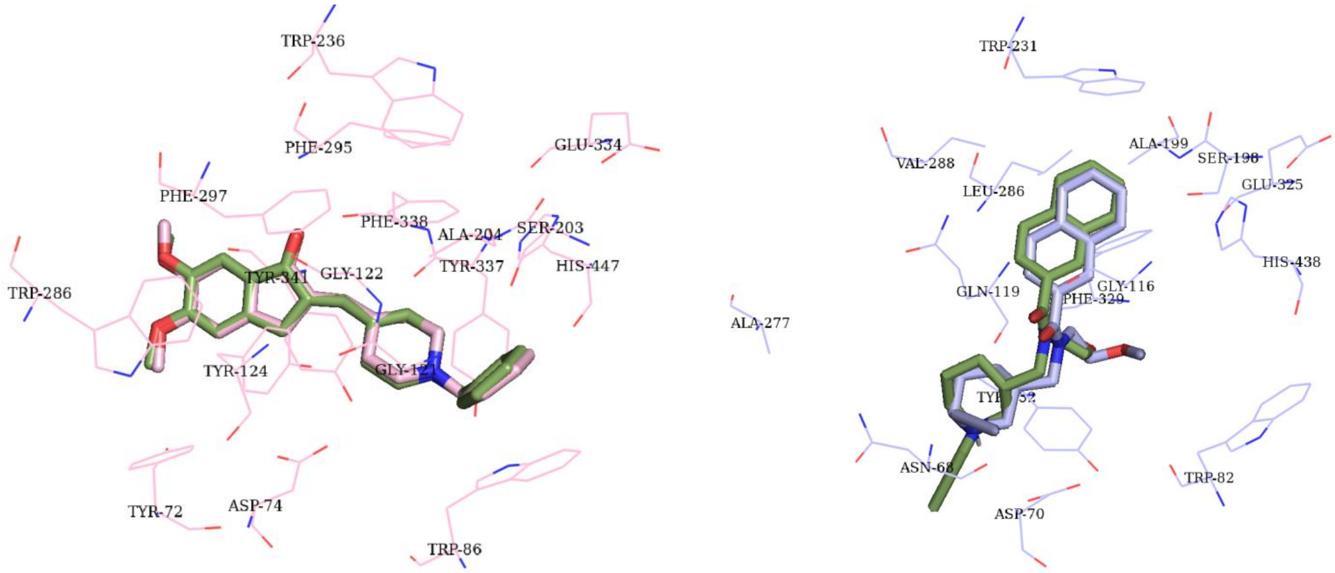

A) B)

Figure S11**.** Self-docking results of (A) *h*AChE in 4EY7 and (B) *h*BChE in 5LKR. Docked poses are highlighted in green.

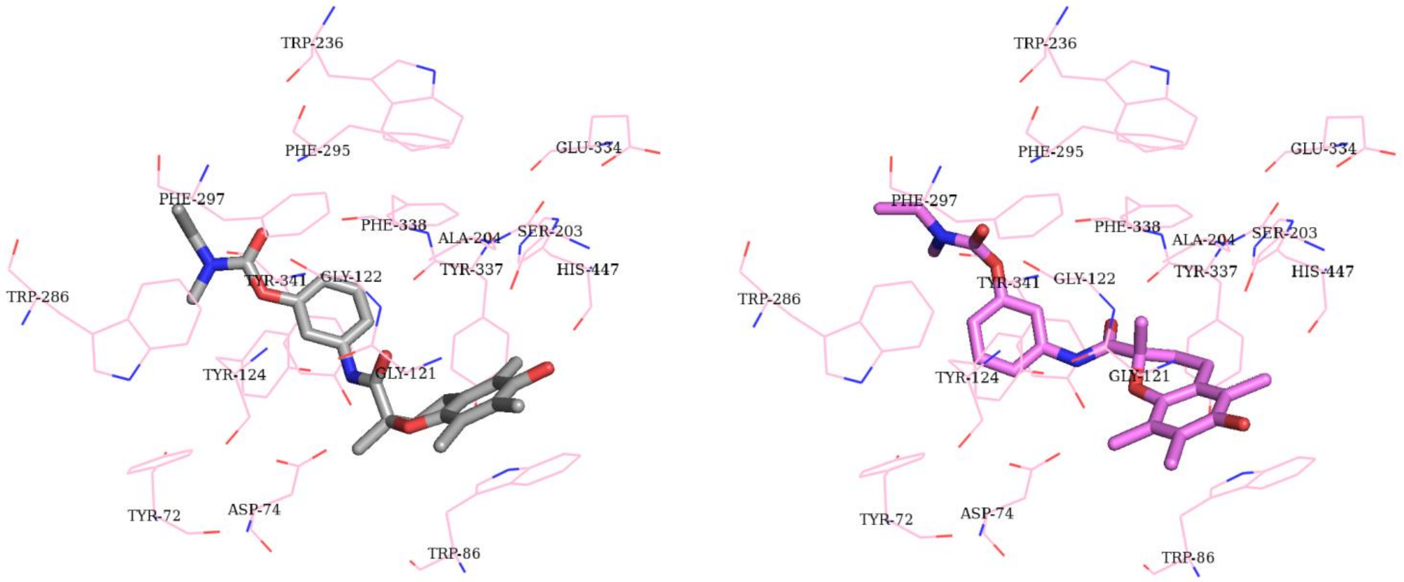

A) B)

Figure S12**.** Predicted binding poses for **4AY2** within the active site of *h*AChE based on the crystal structure with PDB ID: 4EY7: A) R enantiomer (shown in grey); B) S enantiomer (shown in light pink).

3. Post hoc tests related with Table 1

Table S1. Post hoc tests related with EC_{50}, IC_{50} (AChE), IC_{50} (BChE) and % of Aβ self-aggregation inhibition assays; * signifies no statistically significant differences with the reference compound at a 95% confidence level ($p \geq 0.05$).

AA EC_{50} (vs Trolox)		IC_{50} (AChE) (vs rivastigmine)		IC_{50} (BChE) (vs rivastigmine)		% Aβ Self-Agg. Inhib. (vs curcumin) C_L = 40 μM	
**4AY2**	p = 0.0001	**4AY2**	p = 0.0320	**4AY1**	p = 0.0027	**4AY1**	p < 0.0001
**4AY5**	p = 0.0030	**4AY3**	p = 0.0006	**4AY2**	p = 0.0058	**4AY2**	p < 0.0001
**4AY6**	p < 0.00001	**4AY4**	p = 0.0063	**4AY3**	p = 0.0282	**4AY3**	p < 0.0001
**4BY2**	p = 0.0002	**4AY5**	p = 0.0069	**4AY4**	p = 0.0007	**4AY5***	p = 0.1813
		**4AY6**	p = 0.0043	**4AY5**	p = 0.0018	**4AY6***	p = 0.2260
		**4BY2***	p = 0.8849	**4AY6**	p = 0.0147	**4BY1**	p = 0.0018
		**4BY3**	P = 0.0158	**4BY1**	p < 0.0001	**4BY2**	p < 0.0001
				**4BY2**	p = 0.0023	**4BY3**	p < 0.0001
				**4BY3***	p = 0.1063		
				**4CY1**	p = 0.0099		

4. Data of scoring function values

Table S2. Scoring function values of the poses for original ligands (donepezil, 6YC) and the RIV hybrids

Compound	hAChE[1]	hBChE[2]
**4AY1**	-8.03	-7.72
**R4AY2**	-9.44	-10.87
**S4AY2**	-9.61	-9.56
**4AY3**	-8.06	-7.33
**4AY4**	-8.20	-6.52
**4AY5**	-8.00	-7.98
**4AY6**	-8.24	-6.84
**4BY1**	-8.68	-7.95
**R4BY2**	-9.76	-10.06
**S4BY2**	-10.14	-9.58
**4BY3**	-8.72	-9.03
**4CY1**	-7.73	-7.24
Donepezil	-8.66	-
6YC	-	-7.27

[1] MOE; [2] GNINA
